# Molecular and clinical characterization of ANG expression in gliomas and its association with tumor-related immune response

**DOI:** 10.3389/fmed.2023.1044402

**Published:** 2023-10-19

**Authors:** Jin Wang, Aijun Shan, Fei Shi, Qijun Zheng

**Affiliations:** ^1^Department of Emergency, Shenzhen People’s Hospital, The Second Clinical Medical College, Jinan University, Shenzhen, China; ^2^Department of Cardiovascular Surgery, Shenzhen People’s Hospital, The Second Clinical Medical College, Jinan University, Shenzhen, China

**Keywords:** ANG, angiogenin, glioma, immune response, prognosis

## Abstract

**Background:**

Angiogenin (ANG) has been widely reported as a crucial molecular regulator in multiple malignancies. However, its role in gliomagenesis remains unclear. This study aimed to investigate the molecular and clinical characterization of ANG expression at transcriptome level and the association with glioma-related immune response.

**Methods:**

A total of 301 glioma samples with mRNA microarray data (CGGA301) was obtained from the official website of CGGA project for yielding preliminary results, followed by validation in two independent RNAseq datasets, including TCGA with 697 samples and CGGA325 with 325 patients. Moreover, CGGA single-cell RNAseq (scRNAseq) data were analyzed to identify differential and dynamic ANG expression in different cells. Immunohistochemistry was performed to evaluate ANG protein expression across different WHO grades in a tissue microarray (TMA). Figure generation and statistical analysis were conducted using R software.

**Results:**

ANG expression was associated with clinical features, malignant phenotypes, and genomic alterations. Based on significantly correlated genes of ANG, subsequent gene ontology (GO) and gene set enrichment analysis (GSEA) concordantly pointed to the significant association of ANG in immune-related biological processes. Moreover, ANG showed robust correlations with canonical immune checkpoint molecules, including PD1 signaling, CTLA4, TIM3, and B7H3. Gene sets variation analysis (GSVA) found that ANG was particularly associated with activities of macrophages and antigen presentation cells (APCs) in both LGG and GBM across different datasets. Furthermore, the higher-ANG milieu seemed to recruit monocyte–macrophage lineage and dendritic cells into the glioma microenvironment. According to scRNAseq analysis, ANG was mainly expressed by neoplastic cells and tumor-associated macrophages (TAMs) and was correlated with the initiation and progression of tumor cells and the polarization of TAMs. Finally, Kaplan–Meier plots demonstrated that higher expression of ANG was significantly correlated with shorter survival in gliomas. Cox regression analysis further confirmed ANG as an independent predictor of prognosis for gliomas of all three datasets.

**Conclusion:**

ANG is significantly correlated with a range of malignant and aggressive characteristics in gliomas and reveals considerable prognostic value for glioma patients. ANG seems to be primarily associated with immune activities of macrophages and APCs in gliomas. Furthermore, ANG is mainly expressed in neoplastic cells and TAMs and is involved in the initiation and progression of neoplastic cells as well as macrophage polarization.

## Introduction

Over the past several decades, gliomas have become the leading cause of death among central nervous system (CNS) malignancies across different populations worldwide (1). Despite tremendous advances in diagnostic approaches and therapeutic strategies for gliomas, most patients still have dismal prognoses (2). Especially for patients with glioblastoma (GBM), the most lethal type, rarely survive two years after diagnosis (3). The ascending incidence and remarkable lethality of gliomas prompt the urgent need to clarify the molecular mechanisms underlying gliomagenesis. The initiation and progression of glioma is a complex and inter-connected process: it has been proposed that one of the essential driving forces is the immune evasion of tumor cells (2, 4).

Angiogenin (ANG) has been extensively reported across a variety of malignant diseases, including gestational trophoblast neoplasia (5, 6), prostate cancer (7, 8), bladder cancer (9–13), nasopharyngeal carcinoma (14, 15), colorectal cancer (16, 17), hepatocellular carcinoma (18), pancreatic cancer (19), renal cell carcinoma (20), gastric and colon cancer (21), lung cancer (22, 23), breast cancer (24, 25), multiple myeloma (26), lymphoma (27, 28), oral cancer (29), neuroblastoma (30), melanoma (31), cervical cancer (32), and laryngeal carcinoma (33). ANG, also termed RNase5, belongs to the RNase A superfamily, whose family members primarily play roles in ribonucleolytic activity. However, the RNase enzymatic activity of ANG is extremely weak compared to other family members (19). Another important role of ANG is promoting and maintaining the formation of new blood vessels. Consequently, it is more often referred to as angiogenin other than RNase5. Moreover, ANG is supposed to play an essential role in a series of malignant phenotypes, including angiogenesis (23, 24, 26, 30, 34), proliferation (7, 8, 11, 31, 35), migration (7, 25), invasion (32), metastatsis (16), recurrence (33), epithelial-mesenchymal transition (EMT) (18, 22), radiochemotherapy resistance (14, 19, 28), and tumor-associated immunity (9, 17). Several studies have attempted to evaluate the correlation between ANG expression and clinical outcome and have yielded relatively consistent results. A comprehensive summary can be drawn from these results. For patients with malignant tumors, ANG upregulation in their tumor tissues was usually accompanied by a significant unfavorable prognosis (14, 15, 27, 28, 30, 32, 33, 36).

To date, no comprehensive report has been published to demonstrate ANG expression in whole-WHO grade gliomas. Only one previous study (37) described the association between clinicopathological factors and ANG expression in patients with GBM, accounting for approximately 30–40% of all gliomas. To investigate the molecular characterization of ANG expression in whole-grade gliomas, we first obtained and analyzed Chinese Glioma Genome Atlas (CGGA) 301 dataset (CGGA301), including mRNA microarray data of 301 samples of whole-grade gliomas. To further validate the results we found in CGGA301, we took advantage of two other independent RNAseq datasets, including 697 gliomas from The Cancer Genome Atlas (TCGA) network^1^ and 325 gliomas from CGGA325 dataset. It was revealed that the consistency of results across all three cohorts was rather satisfactory. This study represents the first integrative report describing the clinical and molecular characterization of ANG in whole-grade glioma patients.

## Materials and methods

### Data collection

At the transcriptional level, 1,323 glioma samples from three public databases were enrolled and analyzed to generate robust and accurate results regarding the molecular characteristics and clinical significance of ANG in glioma. Respectively, mRNA microarray data of 301 glioma samples (CGGA301 dataset), including clinical information, were downloaded from CGGA (38) website.^2^ Additionally, two other independent glioma cohorts with RNAseq data (RSEM-normalized), including TCGA dataset (39) containing 697 samples^3^ and CGGA325 dataset containing 325 samples, were utilized as the validation sets. In CGGA301 dataset, glioma samples were mainly Chinese people, ranging from WHO grade II to grade IV, and the transcriptome expression data were generated by Agilent Whole Human Genome Array platform. In CGGA325 dataset, samples were also Chinese people, ranging from WHO grade II to grade IV, and the sequencing data were generated by Illumina Hiseq platform. CGGA301 and CGGA325 were collected by the same institution at different times and, therefore, were two independent datasets. In TCGA dataset, samples were mainly caucasian people, ranging from grade II to grade IV, which were detected by high-throughput RNA sequencing technique. Totally, 1,323 glioma samples were enrolled for analysis. Finally, single-cell RNAseq (scRNAseq) expression data of 14 gliomas were also available from CGGA (40) project, in which 6,148 cells were collected from 73 lesion sites. The study flowchart is shown in Supplementary Figure S1.

### Ethics statement

The Ethics Committee of Shenzhen People’s Hospital approved this study. Written informed consent was waived, given the use of deidentified data from public electronic resources. Use of the tissue microarray (TMA) sections was approved by the Ethics Committee of Outdo Biotech Company (Outdo Biotech, Shanghai, China).

### Data preprocessing

For TCGA and CGGA325 datasets, the RSEM-normalized gene expression matrices were log2-transformed prior to data analysis. For CGGA301 microarray dataset, the gene expression matrix was already normalized and centered by CGGA. Lower-grade gliomas (LGGs) and GBMs were separately extracted for analysis in accordance with the 2021 WHO classification criteria for CNS tumors. GBMs were defined as WHO grade IV gliomas with only IDH-wildtype because IDH-mutant grade IV gliomas can no longer be termed GBMs in the latest WHO classification scheme. For survival analysis, Kaplan–Meier curves and Cox regression models excluded patients with missing clinical information and those with overall survival (OS) less than 30-day to exclude the possibility of treatment complication-related mortality. The scRNAseq data were well preprocessed by CGGA project, with both low-quality genes and cells already removed, and the mitochondria gene proportion < 5%.

### Genomic alteration

The somatic genomic alterations of TCGA cohort, including mutations and copy number alterations (CNAs), were obtained from cBioPortal website (41).^4^ In TCGA dataset, mutation and CNA data were available for 626 and 643 patients, respectively. Differential somatic mutations and CNAs were compared between higher- and lower-ANG groups classified by the median ANG expression (ANG median for mutation: 6.087463 (log2-transformed); ANG median for CNA: 6.108524 (log2-transformed)) and were visualized with the oncoplot function provided in ComplexHeatmap package.

### Functional enrichment analysis

Correlation analysis was performed to identify the highly co-expressed genes of ANG in each dataset. Pearson correlation coefficient or Spearman’s rank correlation coefficient was used depending on the satisfaction of the statistical testing assumptions. Genes with correlation coefficients greater than 0.5 and *p*-value less than 0.05 were defined as highly co-expressed genes of ANG. Subsequently, Gene Ontology (GO) analysis of co-expressed genes was performed using DAVID website (42).^5^ The top 7 GO terms were selected and presented by pheatmap package.

### Gene set enrichment analysis

MSigDB Hallmark gene sets were obtained from GSEA database (43).^6^ Genes lists were sorted by correlation coefficients, followed by pre-ranked GSEA analyses with 1,000 perturbations. Enriched gene sets with statistical significance were determined through a normalized enrichment score (NES) greater than 1.00 and a *false discovery rate* (*FDR*) less than 0.25. The top 5 enriched gene sets were presented using the clusterprofiler package (44).

### Gene set variation analysis

A total of 104 genes, which constituted seven immune-related gene signatures (45), each representing a particular immune function, were selected for Gene Sets Variation Analysis (GSVA) (46) analysis, thereby transforming the immune signatures into seven metagenes. Corrgram plots were generated to present intercorrelations based on correlation coefficients between ANG and seven metagenes.

### Cell type enrichment analysis

The cell type composition of immune cells was inferred using XCELL package (47),^7^ which is a reliable and precise method for estimating the cell type composition of a tissue biospecimen from bulk transcriptome data. Results regarding the association of ANG with immune cell composition were visualized using pheatmap.

### Analysis of scRNAseq data

The Seurat package was utilized for scRNAseq analysis. After normalization, top 2000 variable genes were identified by the “vst” method using FindVariableGenes function. Then PCA was performed on the top 2000 variable genes via RunPCA function. Subsequently, we clustered the cells using FindNeighbors and FindClusters functions, with a resolution of 0.1. Finally, UMAP method was performed to visualize the cells, followed by cell annotation with specific cell markers. Four major clusters composed of neoplastic cells (marker: EGFR, PDGFR), macrophages (marker: CD68), T cells (marker: CD3D, CD3E), and oligodendrocytes (marker: MOG) were defined eventually. The ANG expression across different cell types was visualized with Dimplot and VlnPlot. Furthermore, neoplastic cells and macrophages were separately extracted from the Seurat matrix for single-cell pseudotime developmental trajectory analysis with Monocle 2.0 package. Tumor cells and macrophages were consistently divided into three states and were arranged on a pseudotime line.

### Immunohistochemistry of tissue microarray

To examine the protein level of ANG in glioma tissue, we purchased a glioma tissue microarray (TMA) with 125 tissue points (Outdo Biotech, Product ID: HBraG125PG01, Shanghai, China) and performed IHC staining. After antigen retrieval in the PT Link IHC preprocessing system (DAKO, Denmark), the TMA was incubated with an anti-ANG antibody (Proteintech, cat #18302-1-AP; 1:500 dilution) overnight at 4°C. Autostainer Link 48 platform (DAKO, Denmark) and EnVisionTM FLEX+ (K8002, DAKO, Denmark) were employed for secondary antibody binding and color development with diaminobenzidine (DAB), followed by nuclear counterstaining with hematoxylin. The TMA results were captured with Aperio XT Slide Scanner and visualized with Aperio ImageScope software. Finally, each tissue point of the TMA was annotated and analyzed with the algorithm of Positive Pixel Count 2004-08-11 supplied by Aperio ImageScope software (ImageScope v12.4.3.5008; Aperio). The H-score of ANG expression for each tumor tissue point was assessed by a formula involving the positive percentages: score = 1.0 × (% weak positive) + 2.0 × (% positive) + 3.0 × (% strong positive).

### Statistical analysis

All statistical analyses and graphical work were performed using R language together with multiple packages, including dplyr, tidyr, tidyverse, patchwork, ggplot2, ggpubr, ggsci, ggpp, stringr, circlize, ComplexHeatmap, pheatmap, clusterprofiler, xCell, corrgram, GSVA, Seurat, Monocle, survival, survminer, and forestmodel. Gaussian distribution test was performed using before data analysis. When comparing different clinical features and pathological factors between high- and low-ANG groups and when comparing ANG expression levels among different clinicopathological factors, Student’s *t*-test, Wilcoxon test, and chi-square test were used where appropriate. Pearson or Spearman correlation was conducted to evaluate the linear correlation. Kaplan–Meier curves were generated, followed by log-rank tests to evaluate the statistical difference between groups. For whole WHO grades of glioma and the LGG subgroup, the cutoff level of ANG was set at the median value. For the GBM subgroup, ANG expression levels were dichotomized according to the best cutoff values detected by the “surv_cutpoint” function supplied in “survminer” package. Cox regression analysis was performed with coxph function from the survival package and plotted using the forestmodel package. Statistical tests were two-sided, and the significance level was set at *p*-value less than 0.05.

## Results

### ANG is relevant to clinical features and aggressive phenotypes

**Figure 1:**
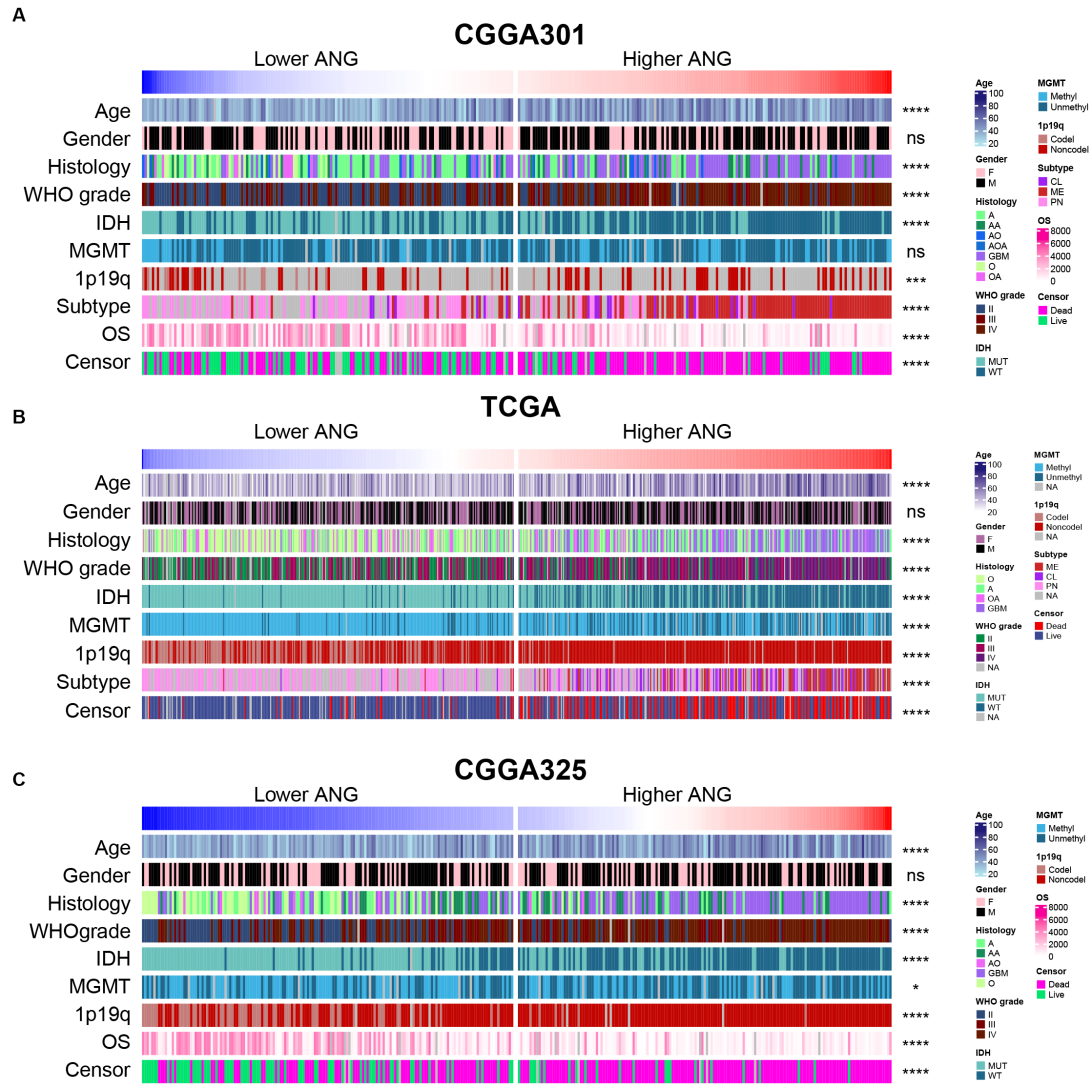
Distribution of clinicopathological characteristics between low- and high-ANG groups. **(A)** The distribution of clinical and pathological characteristics arranged by the increasing ANG expression in CGGA301 dataset. **(B)** The distribution of clinical and pathological characteristics arranged by the increasing ANG expression in TCGA dataset. **(C)** The distribution of clinical and pathological characteristics arranged by the increasing ANG expression in CGGA325 dataset. F, female; M, male; A, astrocytoma; AA, anaplastic astrocytoma; AO, anaplastic oligodendroglioma; AOA, anaplastic oligoastrocytoma; GBM, glioblastoma; MUT, mutation; WT, wildtype; Methyl, methylated; Unmethyl, unmethylated; Codel, codeletion; Noncodel, non-codeletion; CL, classical; ME, mesenchymal; PN, proneural; OS, overall survival; NA, not available. * indicates *p* value <0.05, **indicates *p* value <0.01, *** indicates *p* value <0.001, **** indicates *p* value <0.0001. ns, not significant.

According to ANG expression, patients in each dataset were arranged and divided into lower- and higher- ANG groups. The landscape regarding clinical features and pathological characteristics between both groups is shown in Figure 1. It turned out that higher ANG expression was significantly associated with old age, higher proportion of GBM in histological diagnosis, higher WHO grade, wildtype IDH, non-codeleted 1p/19q, mesenchymal subtype, short OS, and the censor events of death in CGGA301 dataset (Figure 1A). Due to the large sample size, more significant results regarding the clinicopathological factors above-mentioned could be observed in TCGA and CGGA325 datasets (Figures 1B,C). In addition, due to more complete clinical information of TCGA and CGGA325 datasets, patients who had overexpression of ANG tended to be associated with a higher proportion of unmethylated MGMT promoter in these two cohorts.

**Figure 2:**
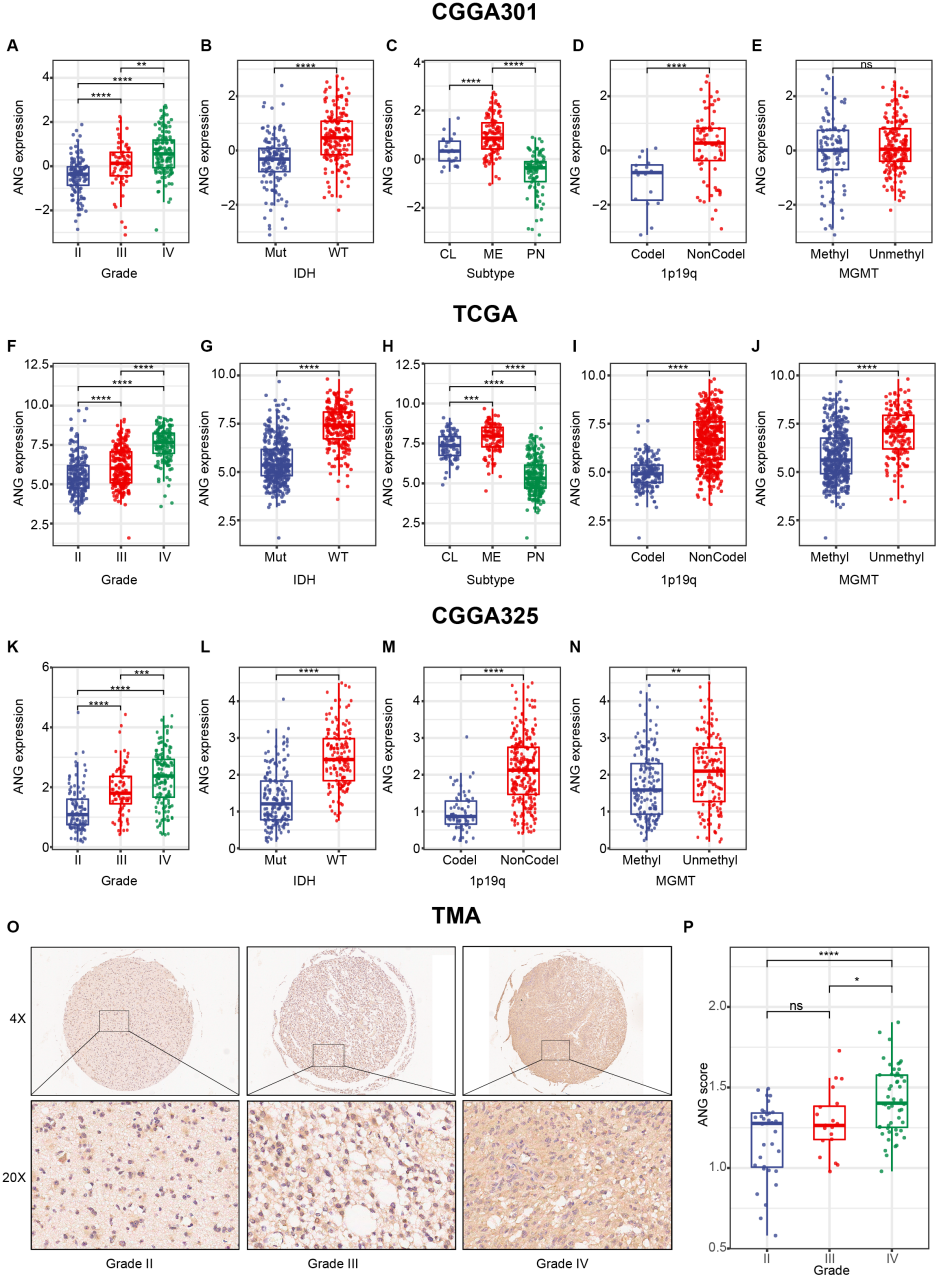
Distribution of ANG expression stratified by pathological characteristics. **(A–E)** Distribution of ANG expression stratified by WHO grade **(A)**, IDH mutation status **(B)**, molecular subtype **(C)**, 1p19q codeletion status **(D)**, and MGMT promoter methylation status **(E)** in CGGA301 dataset. **(F–J)** Distribution of ANG expression stratified by WHO grade **(F)**, IDH mutation status **(G)**, molecular subtype **(H)**, 1p19q codeletion status **(I)**, and MGMT promoter methylation status **(J)** in TCGA dataset. **(K–N)** Distribution of ANG expression stratified by WHO grade **(K)**, IDH mutation status **(L)**, 1p19q codeletion status **(M)**, and MGMT promoter methylation status **(N)** in CGGA325 dataset. **(O)** Representative images of IHC staining for ANG across different WHO grades. **(P)** Comparison of IHC staining intensity (H-score) in different WHO grades of gliomas. Mut, mutation; WT, wildtype; CL, classical; ME, mesenchymal; PN, proneural; Codel, codeletion; NonCodel, non-codeletion; Methyl, methylated; Unmethyl, unmethylated. * indicates *p* value <0.05, **indicates *p* value <0.01, *** indicates *p* value <0.001, **** indicates *p* value <0.0001. ns, not significant.

Furthermore, a series of essential clinical and molecular characteristics were selected to compare ANG expression across different stratifications. Firstly, we compared the differential expression of ANG in different WHO grades. The results agreed very well across the three datasets and showed that ANG expression in higher-grade gliomas was significantly higher than the expression in lower-grade gliomas (Figures 2A,F,K), preliminarily suggesting an association of ANG with malignancy. Secondly, IDH-wildtype gliomas showed a significantly higher ANG expression than those harboring IDH mutation in all datasets (Figures 2B,G,L), further confirming the involvement of ANG in aggressiveness. Thirdly, when patients were grouped according to molecular subtypes, ANG expression was significantly overexpressed in mesenchymal gliomas compared with that in other subtypes (Figures 2C,H). Fourthly, ANG expression was more prevalent in patients with 1p/19q non-codeletion than those with 1p/19q codeletion (Figures 2D,I,M). Fifthly, gliomas with unmethylated MGMT promoter seemed to correspond to an increased level of ANG in contrast to their methylated counterparts (Figures 2E,J,N), though statistical significance was not reached in CGGA301, which might be due to a high number of missing value on MGMT status.

To confirm that ANG expression was also up-regulated at the protein level, we performed IHC staining for ANG on a glioma TMA. Overall, the IHC staining intensity of ANG was moderate in glioma tissues. Following the exclusion of low-quality tissue points, in total, 104 remaining glioma samples were available, including 32 WHO grade II, 20 WHO grade III, and 52 WHO grade IV gliomas. The IHC staining intensity of ANG was measured and compared across different WHO grades (Figures 2O,P). The ANG expression level exhibited an increasing trend with the increasing level of WHO grade, though no statistical significance was observed between grade II and grade III, which might account for the small sample size. Generally, all these results suggested that ANG was closely associated with malignant and aggressive characteristics of glioma based on comprehensive transcriptomic and proteomic analyses.

### Association between ANG and distribution of genomic alteration

**Figure 3:**
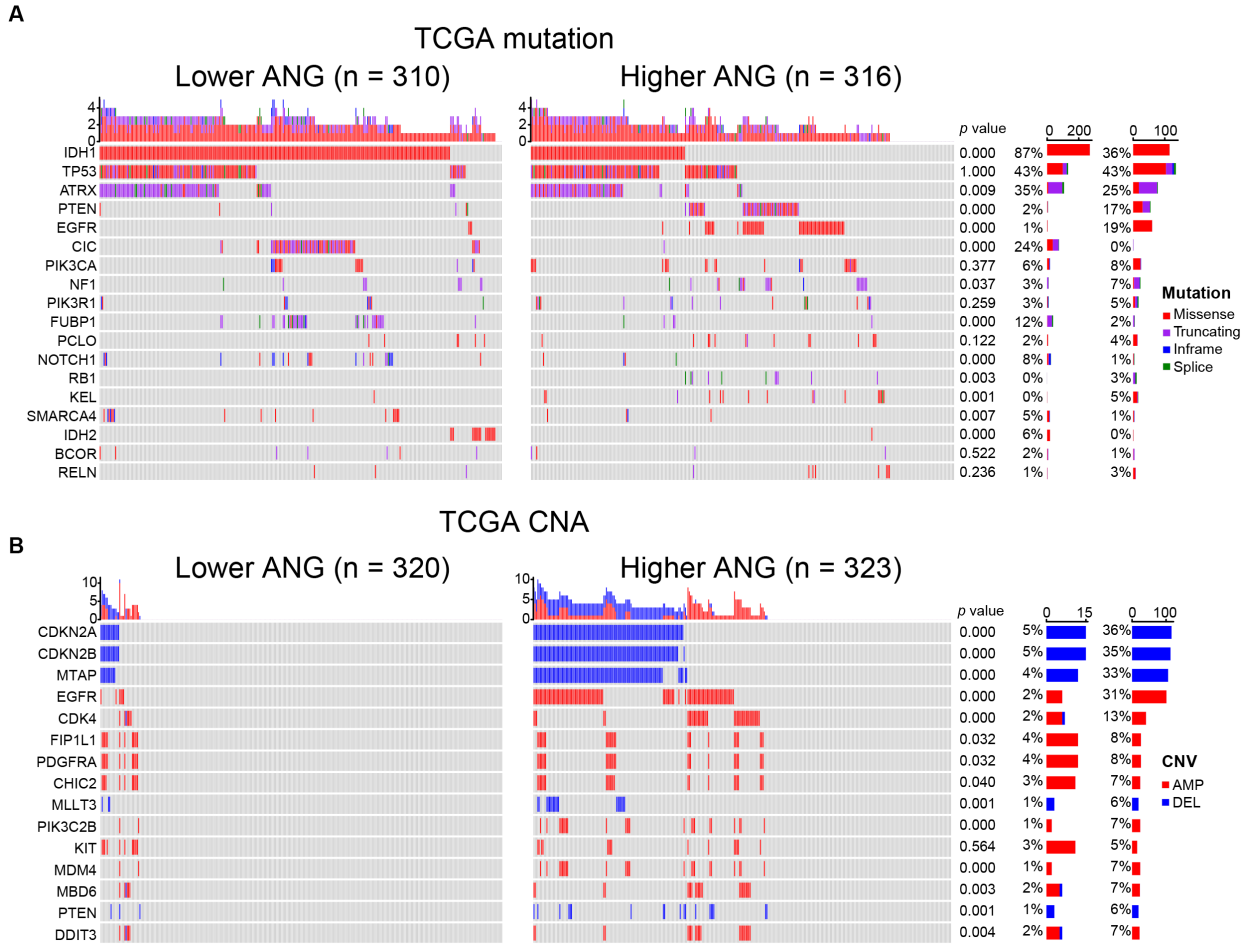
Comparison of genomic alterations between low- and high-ANG groups in TCGA dataset. **(A)** Differential somatic mutations between low- and high-ANG groups. **(B)** Differential copy number variations (CNV) between low- and high-ANG groups. AMP, amplification; DEL, deletion.

To investigate the association between ANG and glioma-specific genomic alterations, somatic mutation profile and CNA data of TCGA were obtained and compared between high- and low-ANG expression groups (Figure 3). Consequently, more frequent mutations of PTEN, EGFR, NF1, RB1, and KEL were observed in the high-ANG group. Meanwhile, the low-ANG group presented a higher proportion of mutations of IDH1, ATRX, CIC, FUBP1, NOTCH1, SMARCA4, and IDH2 (Figure 3A). Moreover, the high ANG expression cohort showed high CNA frequency, with more deletion in tumor-suppressive genes, including CDKN2A, CDKN2B, MTAP, MLLT3, and PTEN, while with more amplification in oncogenic genes, including EGFR, CDK4, FIP1L1, PDGFRA, CHIC2, PIK3C2B, MDM4, MBD6, and DDIT3 (Figure 3B). These findings indicated that gliomas with different ANG expression levels showed different genomic alterations.

### ANG-related biological process

**Figure 4:**
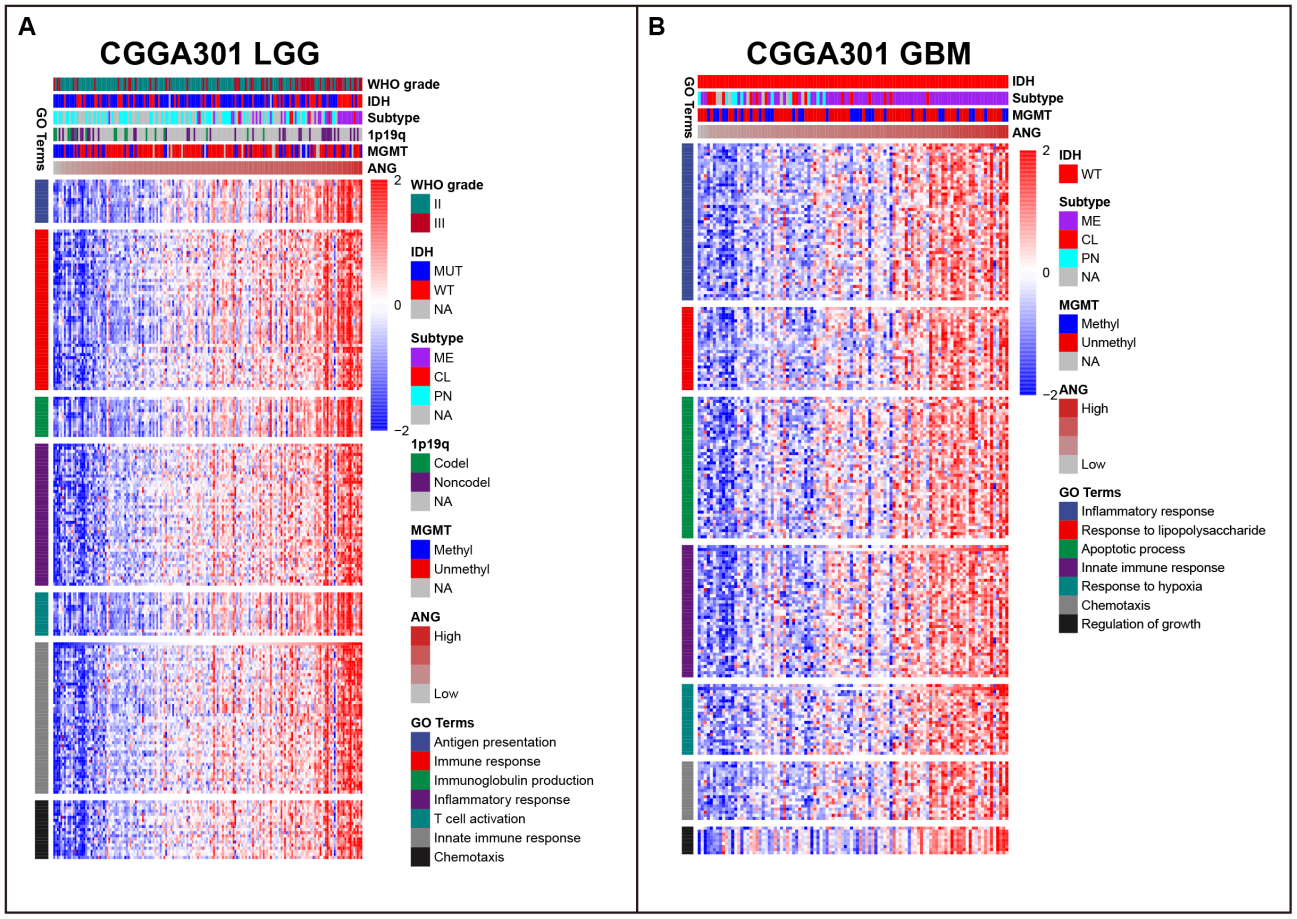
Gene ontology (GO) of genes associated with ANG in CGGA301 dataset. **(A)** Top seven GO terms in CGGA301 lower-grade glioma. **(B)** Top seven GO terms in CGGA301 glioblastoma. LGG, lower grade glioma; GBM, glioblastoma; MUT, mutation; WT, wildtype; CL, classical; ME, mesenchymal; PN, proneural; Codel, codeletion; Noncodel, non-codeletion; Methyl, methylated; Unmethyl, unmethylated; NA, not available.

As shown in Figure 4A, in CGGA301 LGG cohort, ANG co-expressed genes were mainly involved in immune and inflammatory-related biological processes, including antigen presentation, immune response, immunoglobulin production, inflammatory response, T cell activation, innate immune response, and chemotaxis. For CGGA301 GBM cohort (Figure 4B), other than immune-related activities (inflammatory response, response to lipopolysaccharide (LPS), innate immune response, and chemotaxis), ANG was positively associated with more malignant biological processes, including apoptotic process, response to hypoxia, and regulation of growth. Furthermore, GO analysis was performed in TCGA and CGGA325 datasets, in which similar results could be observed. Top seven GO terms for each dataset were listed as follows. TCGA LGG: immune response, innate immune response, inflammatory response, TNF production, antigen presentation, IFN-gamma production, IL-6 production (Supplementary Figure S2A); TCGA GBM: inflammatory response, immune response, antigen presentation, apoptotic process, angiogenesis, autophagy, glycolytic process (Supplementary Figure S2B); CGGA325 LGG: immune response, inflammatory response, angiogenesis, innate immune response, apoptotic process, NF-kappaB signaling, cell adhesion (Supplementary Figure S2C); CGGA325 GBM: inflammatory response, immune response, response to LPS, chemotaxis, apoptotic process, angiogenesis, cellular defense response (Supplementary Figure S2D).

**Figure 5:**
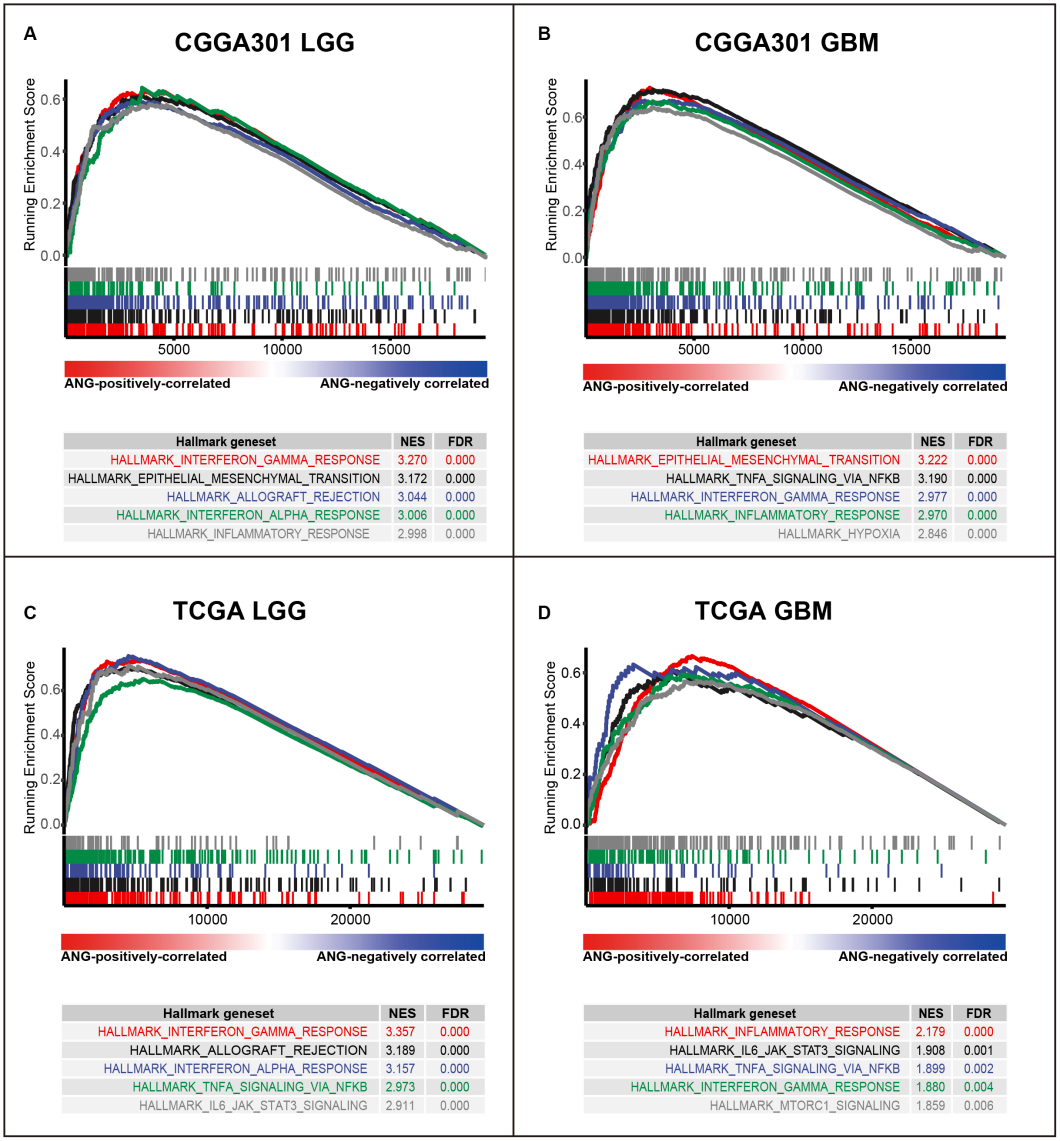
Gene set enrichment analysis (GSEA) of ANG in CGGA301 and TCGA datasets. **(A)** GSEA in CGGA301 lower-grade glioma. **(B)** GSEA in CGGA301 glioblastoma. **(C)** GSEA in TCGA lower-grade glioma. **(D)** GSEA in TCGA glioblastoma. LGG, lower grade glioma; GBM, glioblastoma; NES, normalized enrichment score; *FDR, false discovery rate*.

To further validate the involvement of ANG in immune activities, GSEA analyses were conducted to evaluate the ANG-correlated gene sets. In CGGA301 LGG cohort, the top 5 gene sets enriched in our list of ANG-positively correlated genes were interferon-gamma response (NES = 3.270), epithelial-mesenchymal transition (NES = 3.172), allograft rejection (NES = 3.044), interferon-alpha response (NES = 3.006), and inflammatory response (NES = 2.998) (Figure 5A). Meanwhile, in CGGA301 GBM cohort, the top 5 gene sets were epithelial-mesenchymal transition (NES = 3.222), TNFA signaling via NFKB (NES = 3.190), interferon-gamma response (NES = 2.977), inflammatory response (NES = 2.970), and hypoxia (NES = 2.846) (Figure 5B). These data taken together suggested that ANG-positively-correlated genes (ranked by correlation coefficients) were predominantly enriched in a wide range of immune/inflammatory-related biological processes in both LGG and GBM, which were also validated in TCGA dataset (Figures 5C,D) and CGGA325 dataset (Supplementary Figures S3A,B), further confirming the profound association between ANG and immune response in gliomas.

### ANG is associated with immune checkpoint members

**Figure 6:**
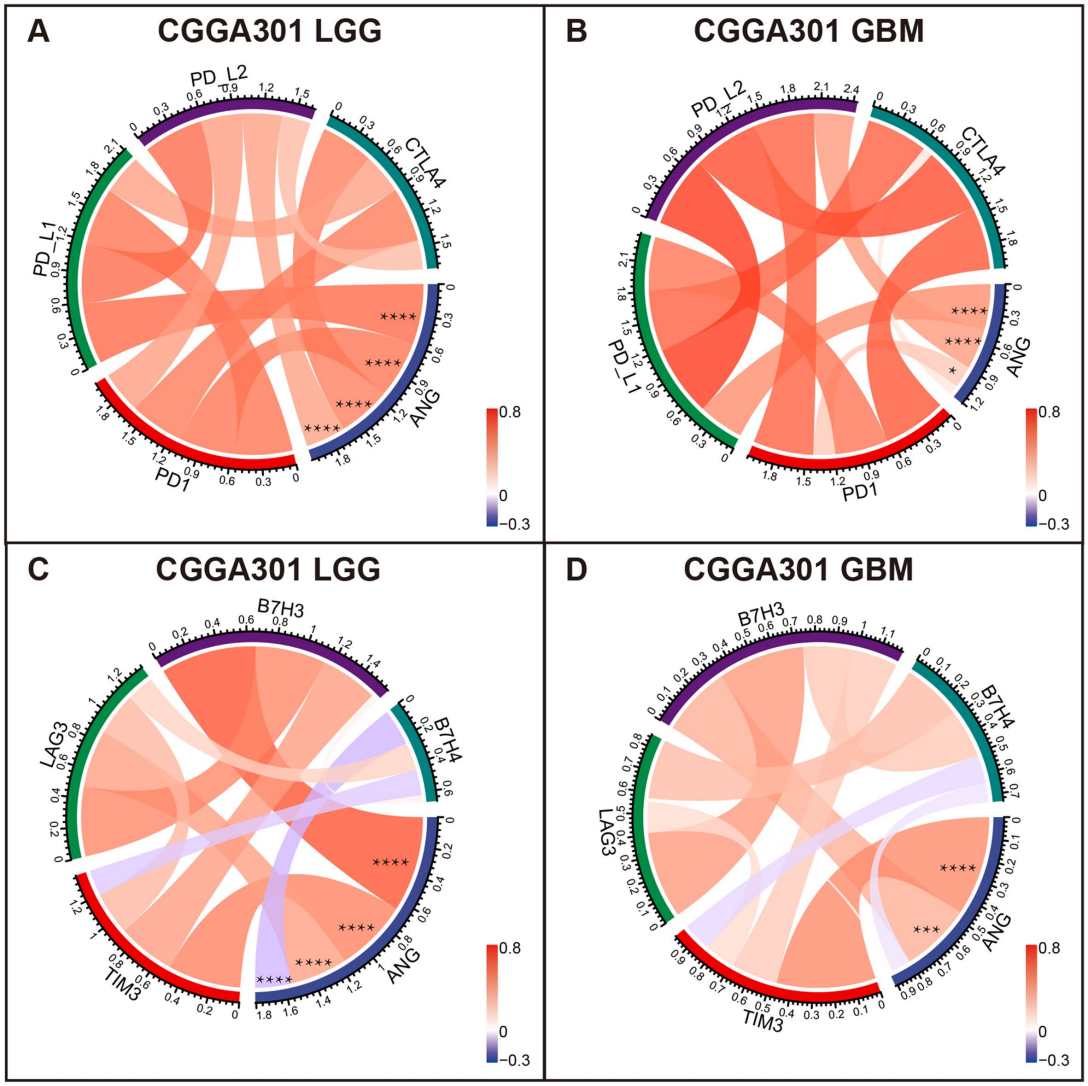
Correlation analysis between ANG and canonical immune checkpoints in CGGA301 dataset. **(A)** Correlation between ANG and pivotal checkpoint members (PD1, PD-L1, PD-L2, and CTLA4) in CGGA301 lower grade glioma. **(B)** Correlation between ANG and pivotal checkpoint members (PD1, PD-L1, PD-L2, and CTLA4) in CGGA301 glioblastoma. **(C)** Correlation between ANG and other checkpoint members (TIM3, LAG3, B7H3, and B7H4) in CGGA301 lower grade glioma. **(D)** Correlation between ANG and other checkpoint members (TIM3, LAG3, B7H3, and B7H4) in CGGA301 glioblastoma. LGG, lower grade glioma; GBM, glioblastoma. * indicates *p* value <0.05, **** indicates *p* value <0.0001.

Correlation tests were performed to evaluate the association between ANG and canonical immune checkpoint members, such as PD1, CTLA4, and TIM3, which have been extensively characterized as promising therapeutic targets for immunotherapy. For LGG cohorts, ANG congruently showed positive correlations with PD1, PD-L1, PD-L2, CTLA4, TIM3, and B7H3 in all three datasets (Figures 6A,C; Supplementary Figures S4A,C,E,G), exhibiting potential synergistic interactions between ANG and these checkpoint members in LGGs. Additional correlation tests were conducted for GBM cohorts of three datasets. ANG also showed robust correlations with these checkpoint members in all datasets (Figures 6B,D; Supplementary Figures S4B,D,F,H), except for CTLA4 in CGGA301 GBM, which might be accounted for signal noise. While the correlations between ANG and LAG3/B7H4 were inconsistent across different datasets, more studies are warranted to draw a definitive conclusion. These results demonstrated that ANG was significantly associated with most of the canonical checkpoint members, including PD1, PD-L1, PD-L2, CTLA4, TIM3, and B7H3, in both LGG and GBM, further validating the close relationship between ANG and glioma immunity.

### ANG-related immune responses

**Figure 7:**
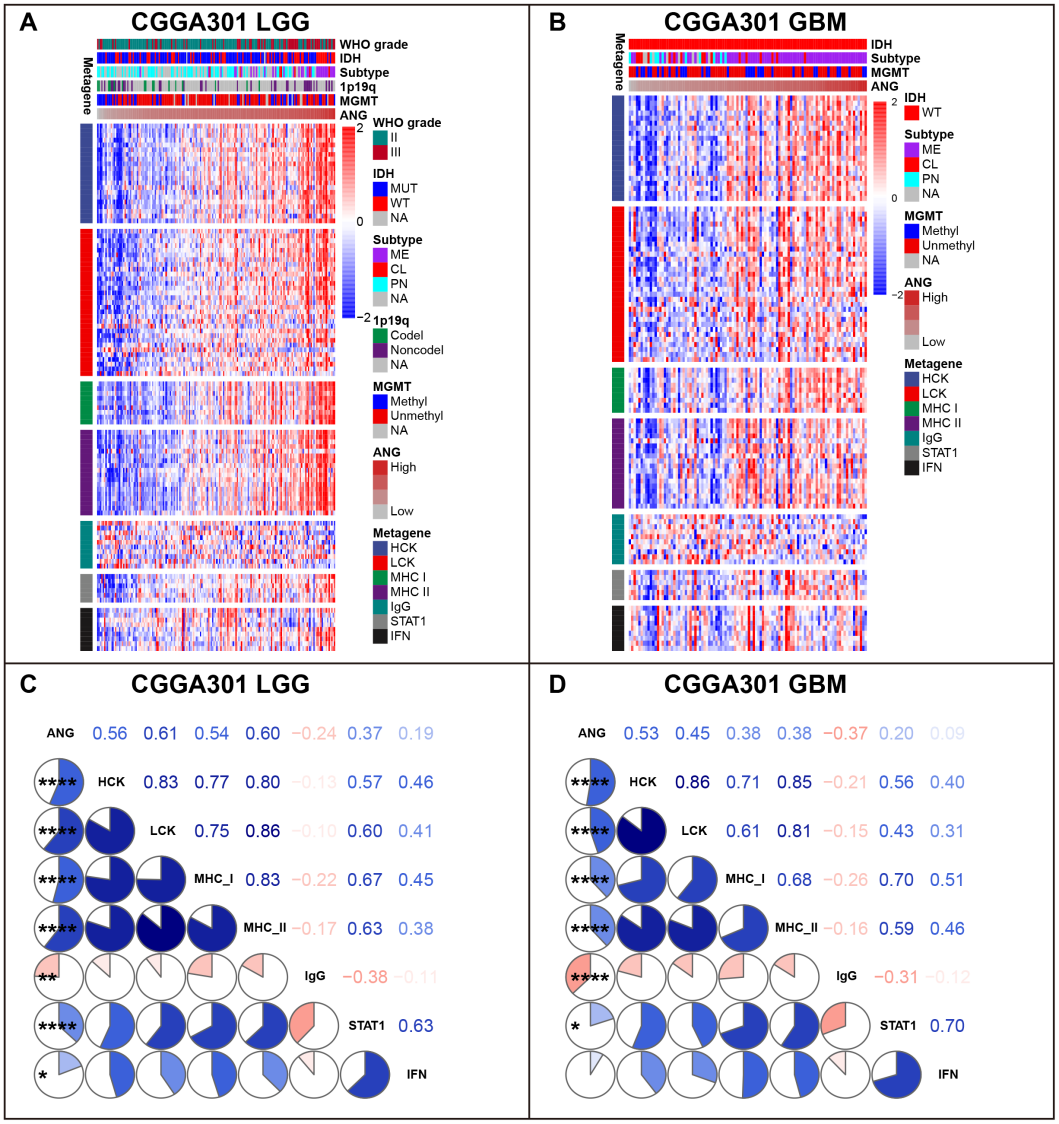
Gene Sets Variation Analysis (GSVA) of ANG-related inflammatory activities in CGGA301 dataset. **(A)** Gene heatmap of different inflammatory activities arranged by ANG expression in CGGA301 lower-grade glioma. **(B)** Gene heatmap of different inflammatory activities arranged by ANG expression in CGGA301 glioblastoma. **(C)** Intercorrelation between ANG and seven metagenes in CGGA301 lower-grade glioma. **(D)** Intercorrelation between ANG and seven metagenes in CGGA301 glioblastoma. LGG, lower grade glioma; GBM, glioblastoma; MUT, mutation; WT, wildtype; CL, classical; ME, mesenchymal; PN, proneural; Codel, codeletion; Noncodel, non-codeletion; Methyl, methylated; Unmethyl, unmethylated; NA, not available; HCK, hemopoietic cell kinase, representing immune activities of the monocyte–macrophage lineage; LCK, lymphocyte-specific kinase, representing T-cell immunity; MHC I, major histocompatibility complex I, representing presentation of intracellular antigens; MHC II, major histocompatibility complex II, representing activities of antigen-presenting cells; IgG, representing B-cell immunity; STAT1, signal transducer and activator of transcription 1, representing interferon signal transduction; IFN, interferon, representing interferon-induction and interferon-response. * indicates *p* value <0.05, **indicates *p* value <0.01, **** indicates *p* value <0.0001.

To understand ANG-related immune responses thoroughly, we selected seven immune signatures (comprising 104 genes), summarized by Rody (45), representing different immune activities and transformed the signatures into seven metagenes via GSVA. In LGG cohorts of three datasets, ANG revealed positive correlations with most immune signatures, except for IgG, which could be defined explicitly as B cell-lineage immune activities (Figure 7A; Supplementary Figures S5A, S6A). Subsequently, we also quantitatively examined the correlations between ANG and seven metagenes using Corrgram plots, in which the results were consistent with our observation in clusters, suggesting the extensive associations between ANG and distinct inflammatory activities in LGGs (Figure 7C; Supplementary Figures S5C, S6C). In GBM cohorts, ANG showed consistently strong correlations with HCK and MHC-I while showing discrepant correlations with the other five metagenes across different datasets (Figures 7B,D; Supplementary Figures S5B,D, S6B,D). These suggested that ANG might be profoundly associated with the monocyte–macrophage lineage activities (HCK) and presentation of intracellular antigens (MHC-I) in GBM.

### ANG is associated with increased monocyte–macrophage and dendritic cells in the tumor microenvironment

**Figure 8:**
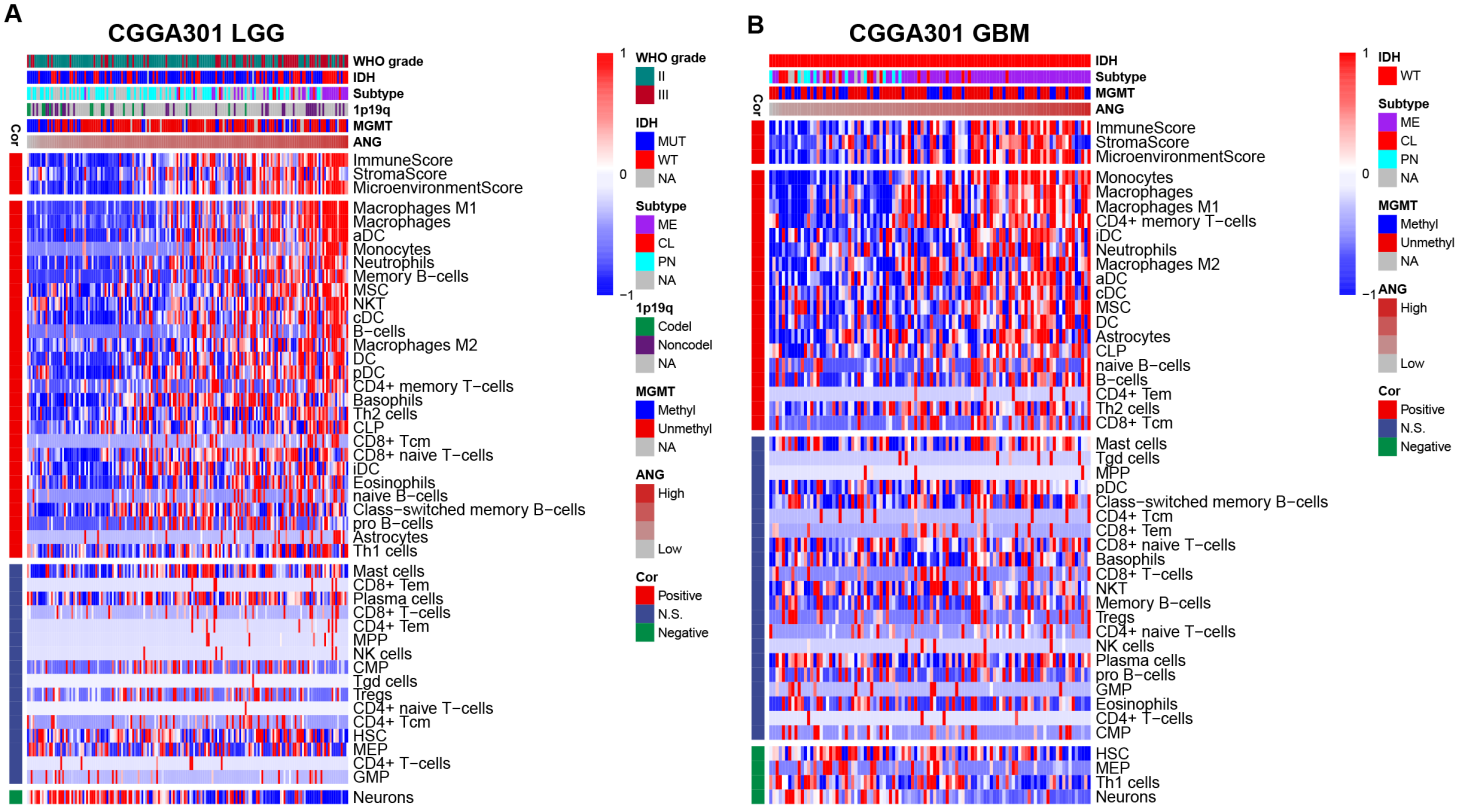
Relationship between ANG and immune cell subpopulations in CGGA301 dataset. **(A)** Correlation between ANG and immune cell subpopulations in CGGA301 lower-grade glioma. **(B)** Correlation between ANG and immune cell subpopulations in CGGA301 glioblastoma. LGG, lower grade glioma; GBM, glioblastoma; MUT, mutation; WT, wildtype; CL, classical; ME, mesenchymal; PN, proneural; Codel, codeletion; Noncodel, non-codeletion; Methyl, methylated; Unmethyl, unmethylated; NA, not available; Cor, correlation; N.S., not significant; DC, dendritic cells; aDC, activated dendritic cells; cDC, conventional dendritic cells; iDC, immature dendritic cells; pDC, plasmacytoid dendritic cells; MSC, mesenchymal stem cells; NKT, natural killer-like T cells; CLP, common lymphoid progenitor; Tcm, central memory T cells; Tem, effector memory T cells; MPP, multipotent progenitors; NK, natural killer; CMP, common myeloid progenitor; Tgd, T gamma delta cells; Tregs, regulatory T cells; HSC, hematopoietic stem cells; MEP, megakaryocyte-erythroid progenitor; GMP, granulocyte-macrophage progenitor.

The xCell analysis (47) was performed to calculate the relative quantification of different immune cells. The results are displayed in Figure 8 and Supplementary Figure S7. In both LGG and GBM cohorts, ANG consistently showed positive correlations with immune, stroma, and microenvironment scores in all three datasets. Moreover, cell composition analysis across different datasets also yielded congruent results. In both LGG and GBM, ANG especially revealed high positive correlations with monocyte–macrophage lineage and dendritic cells (DC), in line with the results from GSVA, in which HCK indicates the macrophage-related activities and MHC-I indicates the antigen presentation process. Altogether, these results showed that higher ANG was tightly associated with multiple types of immune cells, particularly macrophages, and DCs, in the tumor microenvironment, further confirming what we found in GSVA.

### scRNAseq indicates that ANG is mainly expressed by tumor cells and macrophages

**Figure 9:**
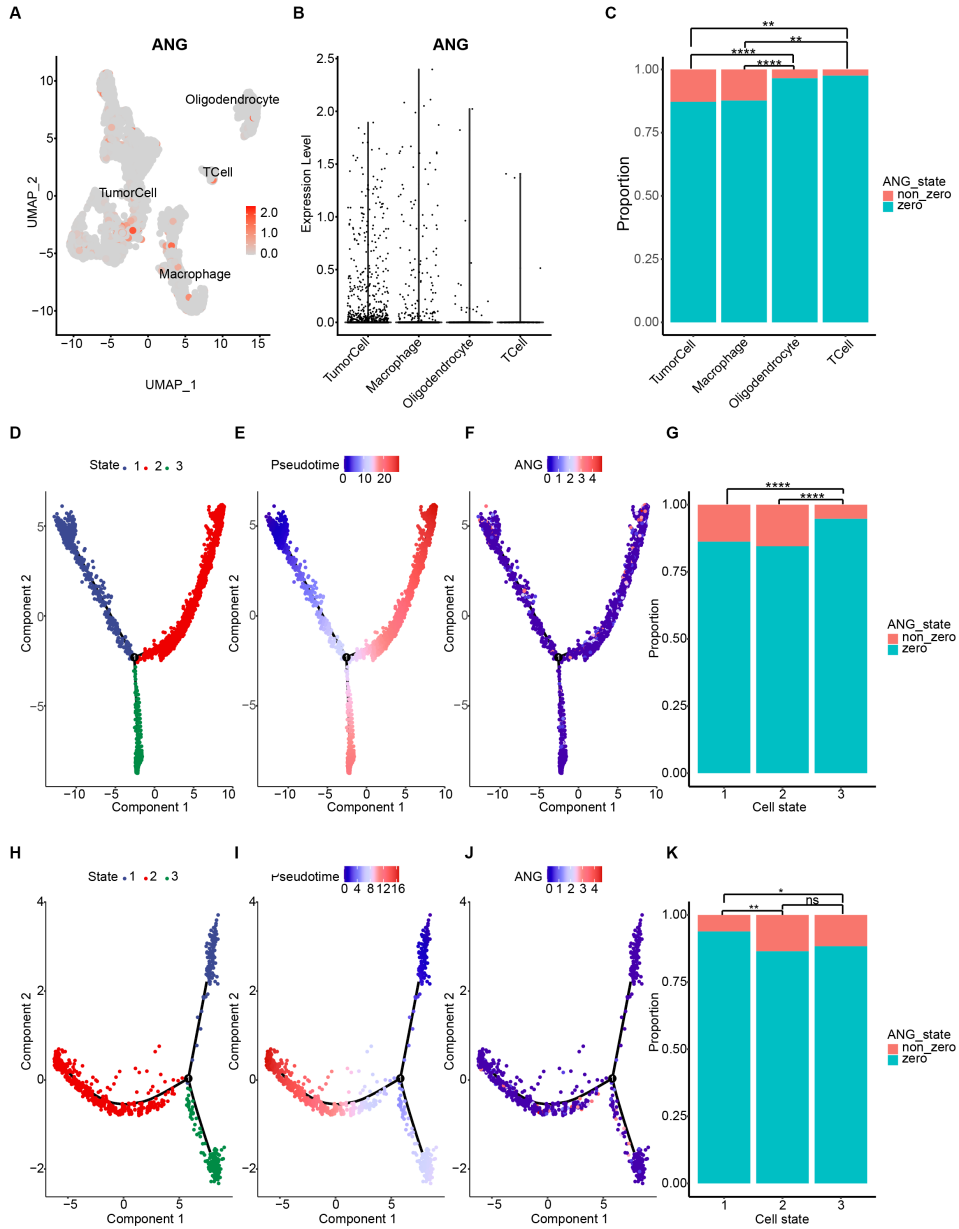
ANG expression on different cell types based on CGGA single-cell RNAseq data. **(A)** Four cell clusters were annotated via cell markers in CGGA scRNAseq data. Dimplot displays ANG distribution across different cell types. **(B)** Vlnplot reveals ANG distribution across different cell types. **(C)** Statistical results of ANG expression status (zero value vs. non-zero value) across different cell types. ANG is mainly expressed by tumor cells and macrophages in glioma. **(D–G)** Single-cell pseudotime developmental trajectory analysis of tumor cells reveals three states. Cells are colored based on states **(D)**, pseudotime **(E)**, and ANG expression **(F)**. State 1 could be identified as early developmental-stage tumor cells, while states 2 and 3 could be defined as mature glioma cells. ANG is more upregulated in early-stage tumor cells and is highly and selectively expressed in a particular branch of mature glioma cells **(G)**. **(H–K)** Single-cell pseudotime developmental trajectory analysis of macrophages reveals three states. Cells are colored based on states **(H)**, pseudotime **(I)**, and ANG expression **(J)**. State 1 could be identified as immature or M0 macrophages, while states 2 and 3 could be defined as polarized macrophages. ANG is significantly upregulated in polarized macrophages rather than immature macrophages **(K)**. * indicates *p* value <0.05, **indicates *p* value <0.01, *** indicates *p* value <0.001, **** indicates *p* value <0.0001. ns, not significant.

To investigate the molecular characteristics of ANG at single-cell resolution, CGGA scRNAseq analysis was performed. As shown in Figures 9A–C, ANG was expressed mainly by neoplastic cells and macrophages. Subsequent single-cell pseudotime developmental trajectory analysis was conducted to demonstrate dynamic changes in ANG expression in neoplastic cells and macrophages. Tumor cells were arranged on trajectories and clustered into three states (Figure 9D), among which state 1 could be identified as early or immature tumor cells, while states 2 and 3 could be defined as mature glioma cells (Figure 9E). ANG expression was significantly higher in state 1 and state 2, suggesting that ANG was more upregulated in early-stage tumor cells and was highly and selectively expressed in a particular branch of mature glioma cells (Figures 9F,G). Moreover, pseudotime developmental trajectory analysis of macrophages also showed three states (Figure 9H), in which state 1 could be identified as immature macrophages or M0 macrophages, while states 2 and 3 were defined as polarized states of macrophages (Figure 9I). ANG was significantly upregulated in polarized macrophages rather than immature macrophages (Figures 9J,K). Our findings suggested that ANG was generated mainly by tumor cells and macrophages and might be associated with glioma initiation and progression and macrophage polarization.

### Higher ANG indicates a worse prognosis

**Figure 10:**
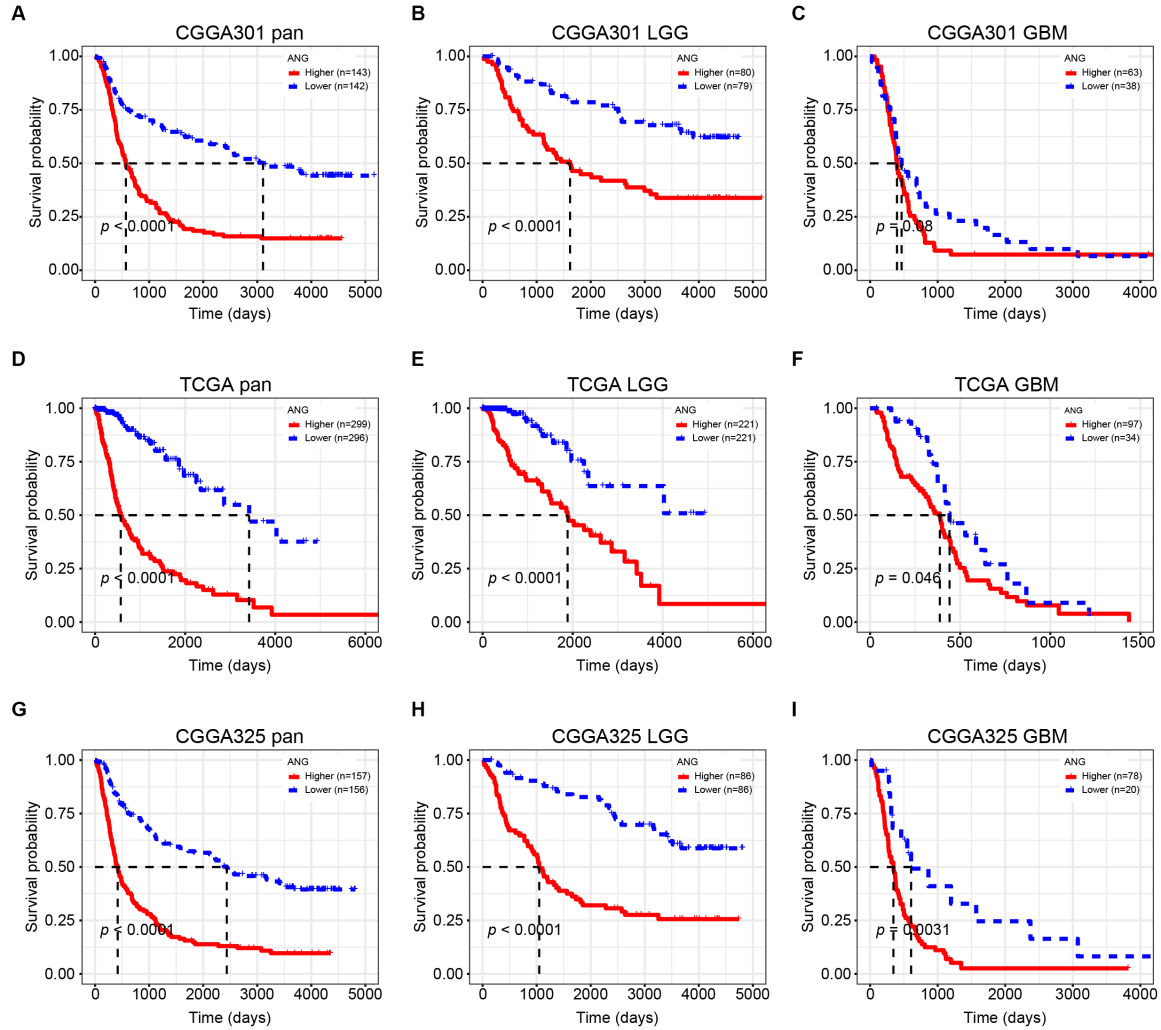
Kaplan–Meier survival curve according to ANG expression. **(A–C)** Survival analysis in CGGA301 dataset. **(D–F)** Survival analysis in TCGA dataset. **(G–I)** Survival analysis in CGGA325 dataset. LGG, lower grade glioma; GBM, glioblastoma.

**Figure 11:**
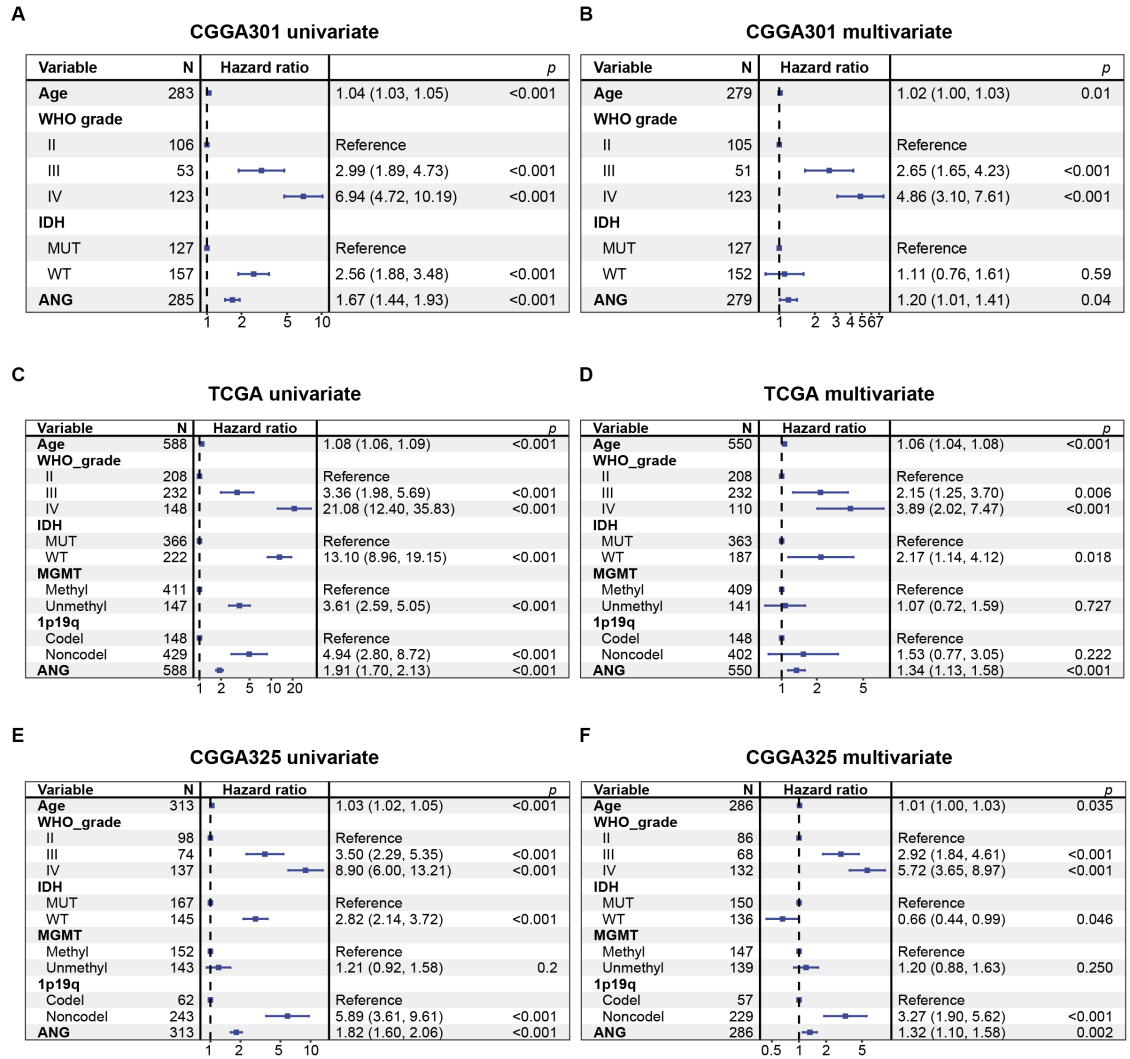
Cox proportional hazards regression analysis of overall survival in glioma patients. **(A)** Univariate Cox regression model in CGGA301 dataset. **(B)** Multivariate Cox regression model in CGGA301 dataset. **(C)** Univariate Cox regression model in TCGA dataset. **(D)** Multivariate Cox regression model in TCGA dataset. **(E)** Univariate Cox regression model in CGGA325 dataset. **(F)** Multivariate Cox regression model in CGGA325 dataset. MUT, mutation; WT, wildtype.

Kaplan–Meier (KM) plots by ANG status are presented in Figure 10. In whole-grade gliomas, patients with higher-ANG expression in their tumors were significantly associated with shorter OS (Figures 10A,D,G). Furthermore, KM plots were performed in both LGG and GBM subgroups. We observed similar patterns that both LGG and GBM patients with higher ANG had universally shorter survival than their counterparts (Figures 10B,C,E,F,H,I) in all datasets, although the difference did not reach statistical significance in CGGA301 GBM, there also showed an apparent trend. Furthermore, the prognostic value of ANG was further evaluated by Cox proportional hazards regression analysis. Multivariate Cox regression analyses showed that the excellent prognostic value of ANG expression was independent of established conventional prognostic factors such as age, WHO grade, and IDH mutation status (CGGA301: *HR* = 1.20, *p* = 0.04; TCGA: *HR* = 1.34, *p* < 0.001; CGGA325: *HR* = 1.32, *p* = 0.002) (Figure 11). In summary, higher ANG was an independent prognostic factor associated with poor clinical outcomes for patients with gliomas.

## Discussion

Gliomas are the most common primary CNS cancers in adults. Notwithstanding the considerable progress in glioma research we have witnessed over the years, some of the most fundamental limitations have not yet been overcome (48). Notably, patients with the diagnosis of GBM, the most aggressive type of glioma, still suffer from dismal prognosis even after undergoing comprehensive therapeutic approaches, including surgery and radiochemotherapy. Over the past few decades, an exciting era of immunotherapy for malignancies has begun, which brings novel cancer therapeutics for these patients, but responses to immunotherapy also vary among different tumors (49). Due to the distinct CNS microenvironment, glioma is supposed to develop and thrive within a highly immunosuppressive or immune-privileged TME (50), in which anti-tumor immunity shows completely different patterns that differ from other neoplasms. Identification of novel immunologic molecules in glioma is hence of high significance.

ANG has been implicated in the pathogenesis of multiple malignant tumors, except for gliomas. To integratively demonstrate and characterize the ANG expression in whole-grade gliomas molecularly and clinically, we conducted this comprehensive analysis based on a total of 1,323 glioma samples from three independent glioma datasets of CGGA301, TCGA, and CGGA325, with which we would like to perform 3-dataset cross-validation to generate more robust results (51–53). Discoveries from CGGA301 as a discovery dataset were further validated in two independent datasets, including TCGA and CGGA325 datasets. The results from three datasets corroborated each other and overall showed good agreement during our analysis.

Clinically, high-ANG was shown to be significantly relevant to a range of malignant clinicopathological characteristics, including elderly age, GBM histology, higher WHO-grade, wildtype IDH, methylated MGMT promoter, non-codeleted 1p19q, mesenchymal molecular subtype, shorter OS, and unfavorable censor outcome, suggesting the close relationship between ANG and aggressive phenotypes of gliomas. Molecularly, the correlation between ANG and glioma malignancy was further explored through the perspective of genomic alterations. High-ANG was significantly associated with high amplification in multiple oncogenic drivers, including EGFR, CDK4, FIP1L1, PDGFRA, CHIC2, PIK3C2B, MDM4, MBD6, and DDIT3 (53), and with more frequent deletions in tumor-suppressing genes like CDKN2A, CDKN2B, MTAP, MLLT3, and PTEN (54). Moreover, high frequent mutations of PTEN, EGFR, NF1, RB1, and KEL, which corresponded to worse prognosis (55), were observed among the higher-ANG group. Meanwhile, mutations in IDH1/2, ATRX, CIC, FUBP1, NOTCH1, and SMARCA4, which are associated with favorable prognosis (56), were more commonly found in the lower-ANG group. Given the profound associations with distinct genomic alterations, ANG is supposed to play crucial roles in glioma genesis, progression, microenvironment remodeling, and drug resistance, further confirming its robust correlation with higher malignancy of gliomas. From the prognosis perspective, KM plots revealed that higher ANG was significantly associated with a shorter OS. Cox proportional hazards regression models of three datasets concordantly revealed that ANG was an independent prognostic predictor for worse survival. Collectively, these results suggested that ANG was upregulated in more aggressive phenotypes and negatively correlated with OS, indicating the tumorigenic potential during malignant progression of gliomas, in line with results from Xia et al. (57), Yang et al. (58), and Hu et al. (37).

Xia et al. (57) performed cytological experiments in U87MG cell, a GBM cell line derived from astrocytoma, and concluded that ANG inhibited GBM cell apoptosis and that ANG could activate NF-kappaB pathway by regulating the expression of FHL3, thereby promoting GBM progression. Our GO analyses in GBMs also revealed a close relationship between ANG and apoptotic process. Yang et al. (58) further established ANG-knockout mice to examine the effect of ANG deficiency on GBM progression and found that ANG deletion prolonged survival time of mice with GBM and decreased invasion, angiogenesis, and proliferation. Consistent with their study, we also observed growth regulation in GO terms of CGGA301 GBM, angiogenesis in TCGA GBM and CGGA325 GBM, epithelial-mesenchymal transition in top 5 GSEA genesets of CGGA301 GBM and CGGA325 GBM. Xia and Yang did perform impressive and excellent experimental studies, and their findings laid the groundwork for evaluating the possible role of ANG in GBM progression. While in our study, other than the biological processes above-mentioned, we would like to highlight the importance of ANG in the tumor-related immune response.

To better understand the involvement of ANG in immune-related biological processes, we conducted in-depth immune-related analysis, including GO, GSEA, correlation analysis with canonical immune checkpoint members, GSVA, immune cell subpopulation analysis, and scRNAseq analysis. First, we performed functional enrichment analysis based on both GO and GSEA algorithms in both LGG and GBM across three datasets. All results congruently pointed out that ANG was profoundly correlated with immune-related biological processes. Second, ANG showed tight associations with most canonical immune checkpoints, such as PD1, CTLA4, and TIM3, suggesting that ANG might interact synergistically with these checkpoint members, all of which have been identified to play a suppressive effect on anti-tumor immunity. Third, GSVA results based on seven immune signatures revealed that ANG was especially correlated with HCK and MHC-I in both LGG and GBM across different datasets, suggesting the robust correlation between ANG and macrophage and antigen-presentation cell-related immune activities (59). Fourth, in cell type composition analysis, ANG expression was positively correlated with immune, stromal, and microenvironment scores. Meanwhile, overexpression of ANG was more inclined to recruit monocyte–macrophage lineage cells and dendritic cells into the glioma TME, further validating what we found in GSVA. Given the profound associations between ANG and a wide range of immune processes that we elaborate above, ANG is supposed to be deeply involved in immune response of gliomas, especially in TAM activities, and to contribute to facilitating the formation of an immunosuppressive microenvironment, immune evasion of neoplastic cells, and tumor progression in gliomas.

Although the involvement of ANG in immune response was not reported in malignancies, several studies reported the immunomodulatory effects of ANG in autoimmune and inflammatory diseases (60–65). In autoimmune diseases, elevated serum ANG was found in patients with inflammatory bowel diseases (IBD) and was associated with distinct clinical features, including lesion location, treatment, smoking habits, and gender (60). Eleftheriadis et al. (61) reported that angiogenin was upregulated and could protect against CD4(+) T-cell apoptosis via inhibition of the T-cell contraction phase in the alloreactive immune response. Park et al. (62) observed that ANG reduced immune-mediated inflammation in rats with endotoxin-induced uveitis and concluded that ANG showed potential for effectively suppressing immune-inflammatory responses *in vivo*. Two other studies in human corneal fibroblast cells (63, 64) pointed out that ANG inhibited TNF-alpha or LPS-induced inflammation via regulating NF-kappaB signaling pathway. Finally, as an inflammation-induced protein, ANG showed a broad spectrum of antibacterial activity against bacteria and fungi, demonstrating the immunoregulatory role during infection-related immune response (65). To sum up, these studies established ANG as a crucial immune-related regulator during particular events, such as autoimmune diseases and infectious diseases.

The applications of scRNAseq technology in molecular characteristics investigation of a specific gene have already been developed (66, 67). Results from scRNAseq revealed that ANG was mainly produced by tumor cells and monocyte–macrophages in glioma TME. scRNAseq pseudotime developmental trajectory analysis identified that ANG was highly expressed in early-stage or immature glioma cells and selectively expressed in a relatively large branch of mature glioma cells, suggesting the regulatory potential of ANG in initiation and progression of particular glioma cells. Moreover, pseudotime trajectory analysis of macrophages showed that ANG was significantly upregulated in polarized macrophages rather than immature macrophages. As a critical component of TME, TAM has been found to play a multifaceted role in glioma initiation and progression (68). Specifically, M2 macrophages have been proven to facilitate tumor aggressiveness, invasion, angiogenesis, as well as immune evasion across different malignant tumors (69). Such results provide the molecular origin contexts for ANG and enable the regulation of glioma-related immune responses.

In our study, ANG was comprehensively analyzed in both LGG and GBM based on three independent glioma cohorts, mainly highlighting the perspectives of the clinicopathological relationship and molecular characterization. Regarding the well-designed studies performed by Hu et al. (37), Yang et al. (58), and Xia et al. (57), they performed seminal and innovative research on ANG in GBM, which laid solid foundations for ANG studies in gliomas. Based on these studies, we emphasized the vital role of ANG in all grades of gliomas and proposed the profound association between ANG and glioma-related immune response, which further extended the results of their studies. Finally, the major limitation of the current study is the lack of biological validation, which is needed to demonstrate ANG as an immune-related target and the precise mechanisms in gliomas.

## Conclusion

ANG is significantly associated with a range of malignant and aggressive characteristics in gliomas and reveals considerable prognostic value for glioma patients. ANG is involved in the immune and inflammatory response of gliomas, expressed mainly in neoplastic cells and macrophages and associated with tumor initiation, progression, and macrophage polarization.

## Data availability statement

The original contributions presented in the study are included in the article/Supplementary material, further inquiries can be directed to the corresponding authors.

## Ethics statement

The studies involving humans were approved by Ethics committee of Shenzhen People’s Hospital. The studies were conducted in accordance with the local legislation and institutional requirements. The ethics committee/institutional review board waived the requirement of written informed consent for participation from the participants or the participants’ legal guardians/next of kin because the use of deidentified data from public electronic resources.

## Author contributions

JW, AS, and FS participated in data acquisition and analysis, and drafted the manuscript. QZ designed this study and revised the manuscript critically. All authors contributed to the article and approved the submitted version.

## Footnotes

^1^ http://cancergenome.nih.gov/

^2^ http://www.cgga.org.cn/

^3^ http://cancergenome.nih.gov/

^4^ http://www.cbioportal.org

^5^ https://david.ncifcrf.gov/

^6^ http://software.broadinstitute.org/

^7^ http://xCell.ucsf.edu/

## References

[1] Ostrom QT Shoaf ML Cioffi G Waite K Kruchko C Wen PY. National-level overall survival patterns for molecularly-defined diffuse glioma types in the United States. Neuro-Oncology. (2022) 25:799–807. doi: 10.1093/neuonc/noac198, PMID: 35994777 PMC10076944

[2] Wang F Zhao F Zhang L Xiong L Mao Q Liu Y. CDC6 is a prognostic biomarker and correlated with immune infiltrates in glioma. Mol Cancer. (2022) 21:153. doi: 10.1186/s12943-022-01623-8, PMID: 35879762 PMC9316328

[3] Yang K Wu Z Zhang H Zhang N Wu W Wang Z. Glioma targeted therapy: insight into future of molecular approaches. Mol Cancer. (2022) 21:39. doi: 10.1186/s12943-022-01513-z, PMID: 35135556 PMC8822752

[4] Yeo AT Rawal S Delcuze B Christofides A Atayde A Strauss L. Single-cell RNA sequencing reveals evolution of immune landscape during glioblastoma progression. Nat Immunol. (2022) 23:971–84. doi: 10.1038/s41590-022-01215-0, PMID: 35624211 PMC9174057

[5] Weng D Han T Dong J Zhang M Mi Y He Y. Angiogenin and MMP-2 as potential biomarkers in the differential diagnosis of gestational trophoblastic diseases. Medicine (Baltimore). (2022) 101:e28768. doi: 10.1097/MD.0000000000028768, PMID: 35119039 PMC8812619

[6] Shaarawy M El-Mallah SY Sheiba M. Angiogenin and gestational trophoblastic tumors, a promising prognostic marker. Clin Chem Lab Med. (2003) 41:306–10. doi: 10.1515/CCLM.2003.049, PMID: 12705339

[7] Marzo T Ferraro G Cucci LM Pratesi A Hansson O Satriano C. Oxaliplatin inhibits angiogenin proliferative and cell migration effects in prostate cancer cells. J Inorg Biochem. (2022) 226:111657. doi: 10.1016/j.jinorgbio.2021.111657, PMID: 34784565

[8] Li S Hu MG Sun Y Yoshioka N Ibaragi S Sheng J. Angiogenin mediates androgen-stimulated prostate cancer growth and enables castration resistance. Mol Cancer Res. (2013) 11:1203–14. doi: 10.1158/1541-7786.MCR-13-0072, PMID: 23851444 PMC3800479

[9] Szymanska B Sawicka E Jurkowska K Matuszewski M Dembowski J Piwowar A. The relationship between interleukin-13 and angiogenin in patients with bladder cancer. J Physiol Pharmacol. (2021) 72:72. doi: 10.26402/jpp.2021.4.13, PMID: 35072652

[10] Peres R Furuya H Pagano I Shimizu Y Hokutan K Rosser CJ. Angiogenin contributes to bladder cancer tumorigenesis by DNMT3b-mediated MMP2 activation. Oncotarget. (2016) 7:43109–23. doi: 10.18632/oncotarget.10097, PMID: 27317771 PMC5190012

[11] Shu J Huang M Tian Q Shui Q Zhou Y Chen J. Downregulation of angiogenin inhibits the growth and induces apoptosis in human bladder cancer cells through regulating AKT/mTOR signaling pathway. J Mol Histol. (2015) 46:157–71. doi: 10.1007/s10735-014-9608-x, PMID: 25564356

[12] Shabayek MI Sayed OM Attaia HA Awida HA Abozeed H. Diagnostic evaluation of urinary angiogenin (ANG) and clusterin (CLU) as biomarker for bladder cancer. Pathol Oncol Res. (2014) 20:859–66. doi: 10.1007/s12253-014-9765-y, PMID: 24696417

[13] Peng Y Li L Huang M Duan C Zhang L Chen J. Angiogenin interacts with ribonuclease inhibitor regulating PI3K/AKT/mTOR signaling pathway in bladder cancer cells. Cell Signal. (2014) 26:2782–92. doi: 10.1016/j.cellsig.2014.08.021 25193113

[14] Guo SS Liang YJ Liu LT Chen QY Wen YF Liu SL. Increased angiogenin expression correlates with radiation resistance and predicts poor survival for patients with nasopharyngeal carcinoma. Front Pharmacol. (2021) 12:627935. doi: 10.3389/fphar.2021.627935 34512316 PMC8427601

[15] Marioni G Koussis H Scola A Maruzzo M Giacomelli L Karahontziti P. Expression of MASPIN and angiogenin in nasopharyngeal carcinoma: novel preliminary clinico-pathological evidence. Acta Otolaryngol. (2010) 130:952–8. doi: 10.3109/00016480903518034, PMID: 20105109

[16] Li S Shi X Chen M Xu N Sun D Bai R. Angiogenin promotes colorectal cancer metastasis via tiRNA production. Int J Cancer. (2019) 145:1395–407. doi: 10.1002/ijc.32245 30828790

[17] Bruno A Bassani B D'Urso DG Pitaku I Cassinotti E Pelosi G. Angiogenin and the MMP9-TIMP2 axis are up-regulated in proangiogenic, decidual NK-like cells from patients with colorectal cancer. FASEB J. (2018) 32:5365–77. doi: 10.1096/fj.201701103R, PMID: 29763380

[18] Hengjuan LV Liu G Kun LI Mingqiu LI Zhang D. Angiogenin regulates epithelial-mesenchymal transition of hepatocellular carcinoma through upregulation of HMGA2. Pharmazie. (2019) 74:301–4. doi: 10.1691/ph.2019.8943 31109401

[19] Wang YN Lee HH Chou CK Yang WH Wei Y Chen CT. Angiogenin/ribonuclease 5 is an EGFR ligand and a serum biomarker for erlotinib sensitivity in pancreatic cancer. Cancer Cell. (2018) 33:e8:752–769.e8. doi: 10.1016/j.ccell.2018.02.012, PMID: 29606349 PMC5893359

[20] Kandori S Kojima T Matsuoka T Yoshino T Sugiyama A Nakamura E. Phospholipase D2 promotes disease progression of renal cell carcinoma through the induction of angiogenin. Cancer Sci. (2018) 109:1865–75. doi: 10.1111/cas.13609, PMID: 29660846 PMC5989877

[21] Sayar I Gokce A Demirtas L Eken H Cimen FK Cimen O. Necl 4 and RNase 5 are important biomarkers for gastric and Colon adenocarcinomas. Med Sci Monit. (2017) 23:2654–9. doi: 10.12659/msm.902648 28561015 PMC5461883

[22] Xu L Yan Y Xue X Li CG Xu ZY Chen HZ. Angiogenin elevates the invasive potential of squamous cell lung carcinoma cells through epithelialmesenchymal transition. Oncol Rep. (2016) 36:2836–42. doi: 10.3892/or.2016.5107, PMID: 27667357

[23] Yuan Y Wang F Liu XH Gong DJ Cheng HZ Huang SD. Angiogenin is involved in lung adenocarcinoma cell proliferation and angiogenesis. Lung Cancer. (2009) 66:28–36. doi: 10.1016/j.lungcan.2008.12.027, PMID: 19423182

[24] He T Qi F Jia L Wang S Wang C Song N. Tumor cell-secreted angiogenin induces angiogenic activity of endothelial cells by suppressing miR-542-3p. Cancer Lett. (2015) 368:115–25. doi: 10.1016/j.canlet.2015.07.036, PMID: 26272182

[25] Dutta S Bandyopadhyay C Bottero V Veettil MV Wilson L Pins MR. Angiogenin interacts with the plasminogen activation system at the cell surface of breast cancer cells to regulate plasmin formation and cell migration. Mol Oncol. (2014) 8:483–507. doi: 10.1016/j.molonc.2013.12.017, PMID: 24457100 PMC5528647

[26] Tsirakis G Pappa CA Kanellou P Stratinaki MA Xekalou A Psarakis FE. Role of platelet-derived growth factor-AB in tumour growth and angiogenesis in relation with other angiogenic cytokines in multiple myeloma. Hematol Oncol. (2012) 30:131–6. doi: 10.1002/hon.1014 21919032

[27] Miyagaki T Sugaya M Suga H Akamata K Ohmatsu H Fujita H. Angiogenin levels are increased in lesional skin and sera in patients with erythrodermic cutaneous T cell lymphoma. Arch Dermatol Res. (2012) 304:401–6. doi: 10.1007/s00403-012-1238-0, PMID: 22526325

[28] Fang S Repo H Joensuu H Orpana A Salven P. High serum angiogenin at diagnosis predicts for failure on long-term treatment response and for poor overall survival in non-Hodgkin lymphoma. Eur J Cancer. (2011) 47:1708–16. doi: 10.1016/j.ejca.2011.02.018, PMID: 21439815

[29] Kishimoto K Yoshida S Ibaragi S Yoshioka N Okui T Hu GF. Hypoxia-induced up-regulation of angiogenin, besides VEGF, is related to progression of oral cancer. Oral Oncol. (2012) 48:1120–7. doi: 10.1016/j.oraloncology.2012.05.009 22694909

[30] Dungwa JV Uparkar U May MT Ramani P. Angiogenin up-regulation correlates with adverse clinicopathological and biological factors, increased microvascular density and poor patient outcome in neuroblastomas. Histopathology. (2012) 60:911–23. doi: 10.1111/j.1365-2559.2012.04176.x, PMID: 22372545

[31] Zhao J Yang Q Yang J Wang J Fan L Wang L. Basic fibroblast growth factor affects the expression of angiogenin and cell proliferation in A375 human melanoma cells. Tumori. (2011) 97:95–103. doi: 10.1177/030089161109700117, PMID: 21528671

[32] Landt S Mordelt K Schwidde I Barinoff J Korlach S Stoblen F. Prognostic significance of the angiogenic factors angiogenin, endoglin and endostatin in cervical cancer. Anticancer Res. (2011) 31:2651–5. PMID: 21778318

[33] Marioni G Marino F Blandamura S D'Alessandro E Giacomelli L Guzzardo V. Neoangiogenesis in laryngeal carcinoma: angiogenin and CD105 expression is related to carcinoma recurrence rate and disease-free survival. Histopathology. (2010) 57:535–43. doi: 10.1111/j.1365-2559.2010.03664.x, PMID: 20955379

[34] Miyake M Goodison S Lawton A Gomes-Giacoia E Rosser CJ. Angiogenin promotes tumoral growth and angiogenesis by regulating matrix metallopeptidase-2 expression via the ERK1/2 pathway. Oncogene. (2015) 34:890–901. doi: 10.1038/onc.2014.2 24561529 PMC4317372

[35] Sadagopan S Veettil MV Chakraborty S Sharma-Walia N Paudel N Bottero V. Angiogenin functionally interacts with p53 and regulates p53-mediated apoptosis and cell survival. Oncogene. (2012) 31:4835–47. doi: 10.1038/onc.2011.648, PMID: 22266868 PMC3337890

[36] Marioni G Staffieri A Savastano M Marino F Giacomelli L Lionello M. Angiogenin expression in head and neck basaloid and conventional squamous cell carcinoma: a site- and stage-matched comparison. J Oral Pathol Med. (2011) 40:55–60. doi: 10.1111/j.1600-0714.2010.00942.x 20923443

[37] Hu JL Luo WJ Wang H. Angiogenin upregulation independently predicts unfavorable overall survival in proneural subtype of glioblastoma. Technol Cancer Res Treat. (2019) 18:153303381984663. doi: 10.1177/1533033819846636 PMC6515846 31072237

[38] Zhao Z Zhang KN Wang Q Li G Zeng F Zhang Y. Chinese glioma genome atlas (CGGA): a comprehensive resource with functional genomic data from Chinese glioma patients. Genomics Proteomics Bioinformatics. (2021) 19:1–12. doi: 10.1016/j.gpb.2020.10.005 33662628 PMC8498921

[39] Cancer Genome Atlas Research N Weinstein JN Collisson EA Mills GB Shaw KR Ozenberger BA. The cancer genome atlas pan-cancer analysis project. Nat Genet. (2013) 45:1113–20. doi: 10.1038/ng.2764, PMID: 24071849 PMC3919969

[40] Yu K Hu Y Wu F Guo Q Qian Z Hu W. Surveying brain tumor heterogeneity by single-cell RNA-sequencing of multi-sector biopsies. Natl Sci Rev. (2020) 7:1306–18. doi: 10.1093/nsr/nwaa099, PMID: 34692159 PMC8289159

[41] Gao J Aksoy BA Dogrusoz U Dresdner G Gross B Sumer SO. Integrative analysis of complex cancer genomics and clinical profiles using the cBioPortal. Sci Signal. (2013) 6:pl1. doi: 10.1126/scisignal.2004088, PMID: 23550210 PMC4160307

[42] Sherman BT Hao M Qiu J Jiao X Baseler MW Lane HC. DAVID: a web server for functional enrichment analysis and functional annotation of gene lists (2021 update). Nucleic Acids Res. (2022) 50:W216–21. doi: 10.1093/nar/gkac194, PMID: 35325185 PMC9252805

[43] Subramanian A Tamayo P Mootha VK Mukherjee S Ebert BL Gillette MA. Gene set enrichment analysis: a knowledge-based approach for interpreting genome-wide expression profiles. Proc Natl Acad Sci U S A. (2005) 102:15545–50. doi: 10.1073/pnas.0506580102, PMID: 16199517 PMC1239896

[44] Yu G Wang LG Han Y He QY. clusterProfiler: an R package for comparing biological themes among gene clusters. OMICS. (2012) 16:284–7. doi: 10.1089/omi.2011.0118 22455463 PMC3339379

[45] Rody A Holtrich U Pusztai L Liedtke C Gaetje R Ruckhaeberle E. T-cell metagene predicts a favorable prognosis in estrogen receptor-negative and HER2-positive breast cancers. Breast Cancer Res. (2009) 11:R15. doi: 10.1186/bcr2234 19272155 PMC2688939

[46] Hanzelmann S Castelo R Guinney J. GSVA: gene set variation analysis for microarray and RNA-seq data. BMC Bioinformatics. (2013) 14:7. doi: 10.1186/1471-2105-14-7 23323831 PMC3618321

[47] Aran D Hu Z Butte AJ. xCell: digitally portraying the tissue cellular heterogeneity landscape. Genome Biol. (2017) 18:220. doi: 10.1186/s13059-017-1349-1 29141660 PMC5688663

[48] Venkataramani V Yang Y Schubert MC Reyhan E Tetzlaff SK Wissmann N. Glioblastoma hijacks neuronal mechanisms for brain invasion. Cells. (2022) 185:e31:2899–2917.e31. doi: 10.1016/j.cell.2022.06.054, PMID: 35914528

[49] Crunkhorn S. T cell atlas reveals route to glioma immunotherapy. Nat Rev Drug Discov. (2021) 20:261. doi: 10.1038/d41573-021-00038-2 33654224

[50] Mathewson ND Ashenberg O Tirosh I Gritsch S Perez EM Marx S. Inhibitory CD161 receptor identified in glioma-infiltrating T cells by single-cell analysis. Cells. (2021) 184:e26:1281–1298.e26. doi: 10.1016/j.cell.2021.01.022, PMID: 33592174 PMC7935772

[51] Zhou H Meng M Wang Z Zhang H Yang L Li C. The role of m5C-related lncRNAs in predicting overall prognosis and regulating the lower grade glioma microenvironment. Front Oncol. (2022) 12:814742. doi: 10.3389/fonc.2022.814742, PMID: 35372082 PMC8971304

[52] Tian Y Liu H Zhang C Liu W Wu T Yang X. Comprehensive analyses of ferroptosis-related alterations and their prognostic significance in glioblastoma. Front Mol Biosci. (2022) 9:904098. doi: 10.3389/fmolb.2022.904098, PMID: 35720126 PMC9204216

[53] Jin Y Wang Z Xiang K Zhu Y Cheng Y Cao K. Comprehensive development and validation of gene signature for predicting survival in patients with glioblastoma. Front Genet. (2022) 13:900911. doi: 10.3389/fgene.2022.900911, PMID: 36035145 PMC9399759

[54] Varn FS Johnson KC Martinek J Huse JT Nasrallah MP Wesseling P. Glioma progression is shaped by genetic evolution and microenvironment interactions. Cells. (2022) 185:e16:2184–2199.e16. doi: 10.1016/j.cell.2022.04.038, PMID: 35649412 PMC9189056

[55] Mao M Chu Q Lou Y Lv P Wang LJ. RNA N1-methyladenosine regulator-mediated methylation modification patterns and heterogeneous signatures in glioma. Front Immunol. (2022) 13:948630. doi: 10.3389/fimmu.2022.948630, PMID: 35936006 PMC9354098

[56] Li S Zhang N Liu S Zhang H Liu J Qi Y. ITGA5 is a novel oncogenic biomarker and correlates with tumor immune microenvironment in gliomas. Front Oncol. (2022) 12:844144. doi: 10.3389/fonc.2022.844144 35371978 PMC8971292

[57] Xia W Fu W Cai X Wang M Chen H Xing W. Angiogenin promotes U87MG cell proliferation by activating NF-kappaB signaling pathway and downregulating its binding partner FHL3. PLoS One. (2015) 10:e0116983. doi: 10.1371/journal.pone.0116983, PMID: 25659096 PMC4320115

[58] Yang H Yuan L Ibaragi S Li S Shapiro R Vanli N. Angiogenin and plexin-B2 axis promotes glioblastoma progression by enhancing invasion, vascular association, proliferation and survival. Br J Cancer. (2022) 127:422–35. doi: 10.1038/s41416-022-01814-6, PMID: 35418212 PMC9345892

[59] Yang Y Lv W Xu S Shi F Shan A Wang J. Molecular and clinical characterization of LIGHT/TNFSF14 expression at transcriptional level via 998 samples with brain glioma. Front Mol Biosci. (2021) 8:567327. doi: 10.3389/fmolb.2021.567327, PMID: 34513918 PMC8430338

[60] Oikonomou KA Kapsoritakis AN Kapsoritaki AI Manolakis AC Tiaka EK Tsiopoulos FD. Angiogenin, angiopoietin-1, angiopoietin-2, and endostatin serum levels in inflammatory bowel disease. Inflamm Bowel Dis. (2011) 17:963–70. doi: 10.1002/ibd.21410 20629092

[61] Eleftheriadis T Pissas G Sounidaki M Antoniadis N Antoniadi G Liakopoulos V. Angiogenin is upregulated during the alloreactive immune response and has no effect on the T-cell expansion phase, whereas it affects the contraction phase by inhibiting CD4(+) T-cell apoptosis. Exp Ther Med. (2016) 12:3471–5. doi: 10.3892/etm.2016.3786 27882181 PMC5103843

[62] Park J Kim JT Lee SJ Kim JC. The anti-inflammatory effects of angiogenin in an endotoxin induced uveitis in rats. Int J Mol Sci. (2020) 21:21. doi: 10.3390/ijms21020413, PMID: 31936482 PMC7014170

[63] Lee SH Kim KW Min KM Kim KW Chang SI Kim JC. Angiogenin reduces immune inflammation via inhibition of TANK-binding kinase 1 expression in human corneal fibroblast cells. Mediat Inflamm. (2014) 2014:861435:1–12. doi: 10.1155/2014/861435 PMC4016892 24860242

[64] Lee SH Kim KW Joo K Kim JC. Angiogenin ameliorates corneal opacity and neovascularization via regulating immune response in corneal fibroblasts. BMC Ophthalmol. (2016) 16:57. doi: 10.1186/s12886-016-0235-z 27356868 PMC4926301

[65] Hooper LV Stappenbeck TS Hong CV Gordon JI. Angiogenins: a new class of microbicidal proteins involved in innate immunity. Nat Immunol. (2003) 4:269–73. doi: 10.1038/ni888, PMID: 12548285

[66] Chen B Zhou X Yang L Zhou H Meng M Wu H. Glioma stem cell signature predicts the prognosis and the response to tumor treating fields treatment. CNS Neurosci Ther. (2022) 28:2148–62. doi: 10.1111/cns.13956, PMID: 36070228 PMC9627385

[67] Chen B Zhou X Yang L Zhou H Meng M Zhang L. A cuproptosis activation scoring model predicts neoplasm-immunity interactions and personalized treatments in glioma. Comput Biol Med. (2022) 148:105924. doi: 10.1016/j.compbiomed.2022.105924 35964468

[68] Liu Y Shi Y Wu M Liu J Wu H Xu C. Hypoxia-induced polypoid giant cancer cells in glioma promote the transformation of tumor-associated macrophages to a tumor-supportive phenotype. CNS Neurosci Ther. (2022) 28:1326–38. doi: 10.1111/cns.13892, PMID: 35762580 PMC9344088

[69] Yang T Hu Y Miao J Chen J Liu J Cheng Y. A BRD4 PROTAC nanodrug for glioma therapy via the intervention of tumor cells proliferation, apoptosis and M2 macrophages polarization. Acta Pharm Sin B. (2022) 12:2658–71. doi: 10.1016/j.apsb.2022.02.009, PMID: 35755286 PMC9214068

