# Supplementary material for: Molecular and clinical characterization of ANG expression in gliomas and its association with tumor-related immune response
Source: Front Med (Lausanne). 2023 Oct 19;10:1044402. doi: 10.3389/fmed.2023.1044402 (PMC10621067; doi:10.3389/fmed.2023.1044402)
Supplement: Supplementary file 1 [file Data_Sheet_1.docx]

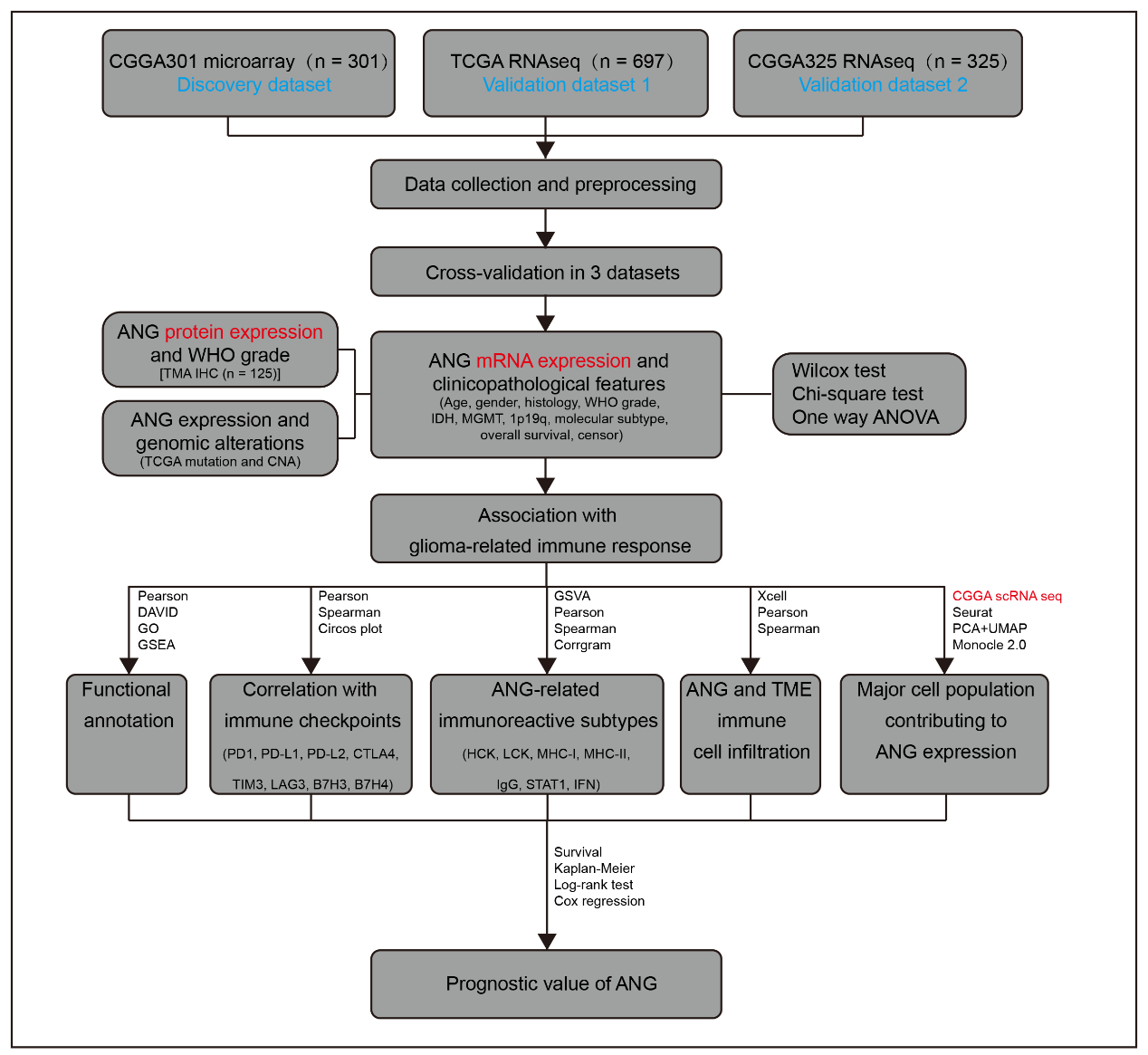


**Figure S1. Flowchart of the study.**

**
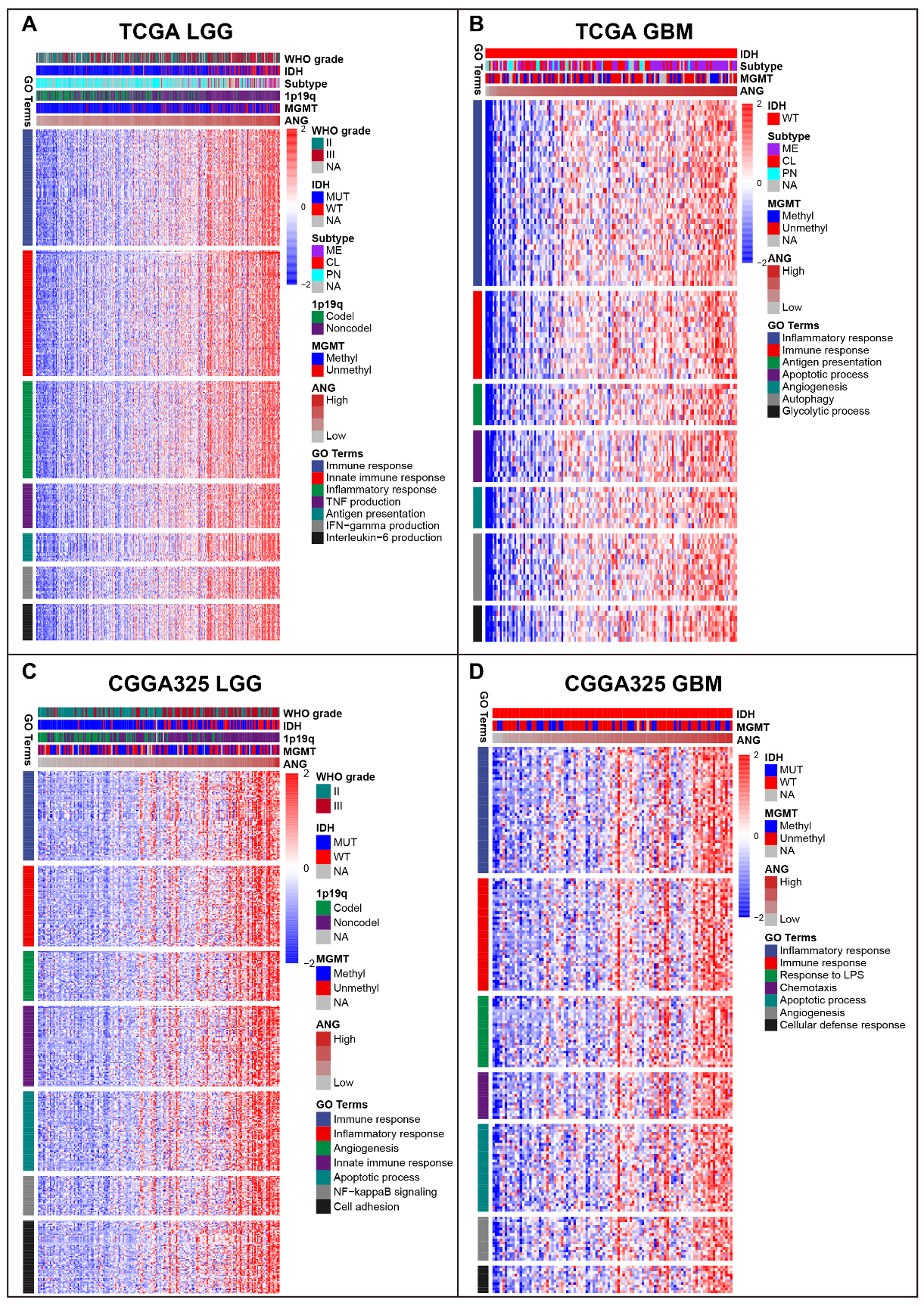
**

**Figure S2.** Gene ontology (GO) of genes associated with ANG in TCGA and CGGA325 dataset. (A) Top seven GO terms in TCGA lower-grade glioma. (B) Top seven GO terms in TCGA glioblastoma. (C) Top seven GO terms in CGGA325 lower-grade glioma. (D) Top seven GO terms in CGGA325 glioblastoma. LGG (Lower grade glioma), GBM (Glioblastoma), MUT (Mutation), WT (Wildtype), CL (Classical), ME (Mesenchymal), PN (Proneural), Codel (Codeletion), Noncodel (Non-codeletion), Methyl (Methylated), Unmethyl (Unmethylated), NA (Not available).


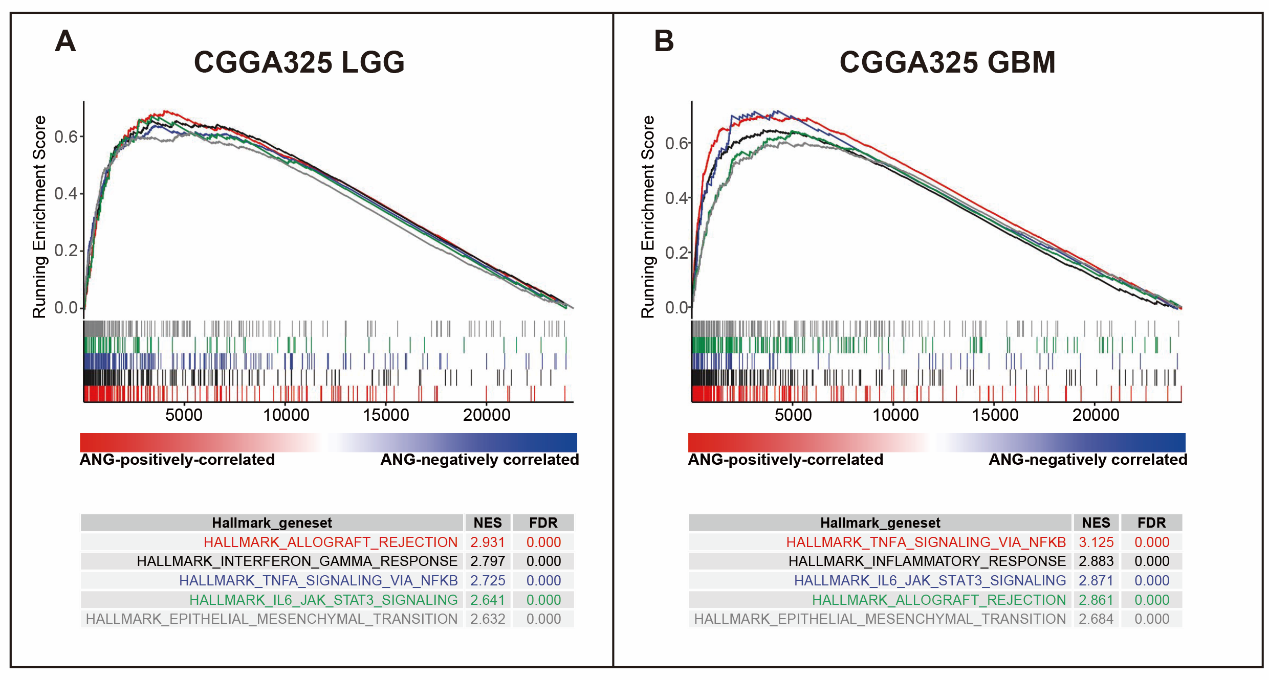


**Figure S3.** Gene set enrichment analysis (GSEA) of ANG in CGGA325 dataset. (A) GSEA in CGGA325 lower-grade glioma. (B) GSEA in CGGA325 glioblastoma. LGG (Lower grade glioma), GBM (Glioblastoma), NES (Normalized enrichment score), *FDR* (*False discovery rate*).

**
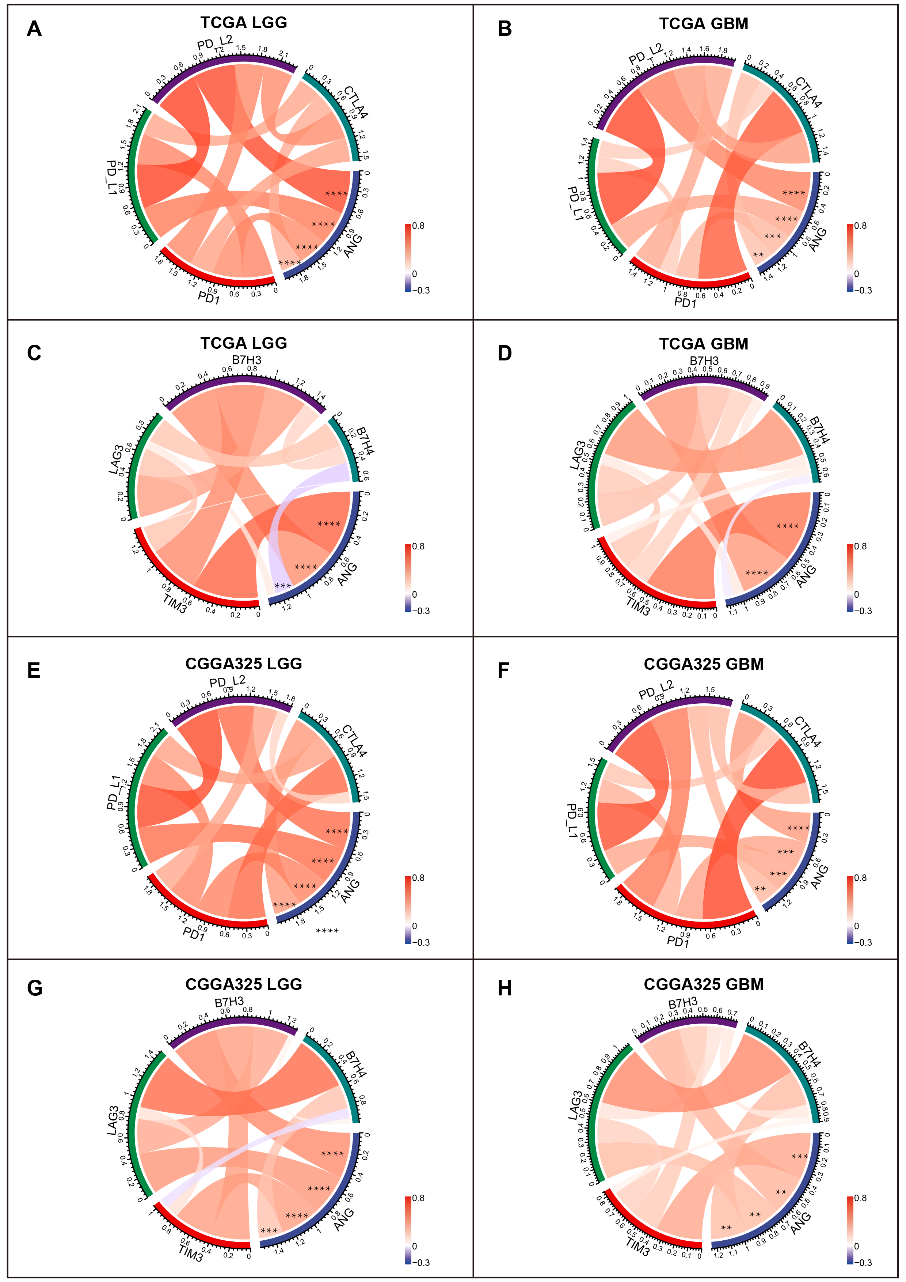
**

**Figure S4.** Correlation analysis between ANG and canonical immune checkpoints in TCGA and CGGA325 datasets. (A) Correlation between ANG and pivotal checkpoint members (PD1, PD-L1, PD-L2, and CTLA4) in TCGA lower grade glioma. (B) Correlation between ANG and pivotal checkpoint members (PD1, PD-L1, PD-L2, and CTLA4) in TCGA glioblastoma. (C) Correlation between ANG and other checkpoint members (TIM3, LAG3, B7H3, and B7H4) in TCGA lower grade glioma. (D) Correlation between ANG and other checkpoint members (TIM3, LAG3, B7H3, and B7H4) in TCGA glioblastoma. (E) Correlation between ANG and pivotal checkpoint members (PD1, PD-L1, PD-L2, and CTLA4) in CGGA325 lower grade glioma. (F) Correlation between ANG and pivotal checkpoint members (PD1, PD-L1, PD-L2, and CTLA4) in CGGA325 glioblastoma. (G) Correlation between ANG and other checkpoint members (TIM3, LAG3, B7H3, and B7H4) in CGGA325 lower grade glioma. (H) Correlation between ANG and other checkpoint members (TIM3, LAG3, B7H3, and B7H4) in CGGA325 glioblastoma. LGG (Lower grade glioma), GBM (Glioblastoma). * indicates *p* value < 0.05, **indicates *p* value < 0.01, *** indicates *p* value < 0.001, **** indicates *p* value < 0.0001.

**
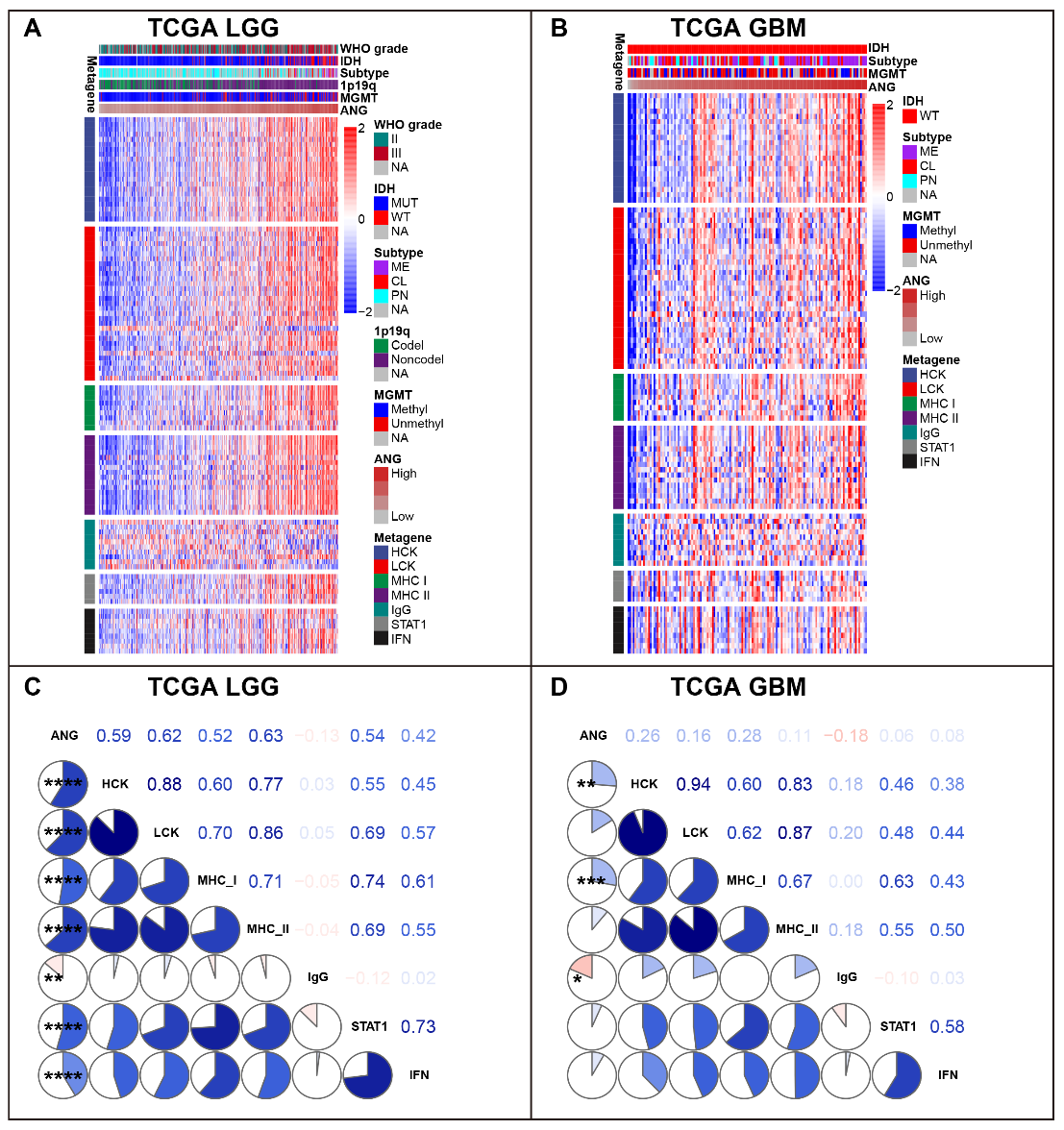
**

**Figure S5.** Gene Sets Variation Analysis (GSVA) of ANG-related inflammatory activities in TCGA dataset. (A) Gene heatmap of different inflammatory activities arranged by ANG expression in TCGA lower-grade glioma. (B) Gene heatmap of different inflammatory activities arranged by ANG expression in TCGA glioblastoma. (C) Intercorrelation between ANG and seven metagenes in TCGA lower-grade glioma. (D) Intercorrelation between ANG and seven metagenes in TCGA glioblastoma. LGG (Lower grade glioma), GBM (Glioblastoma), MUT (Mutation), WT (Wildtype), CL (Classical), ME (Mesenchymal), PN (Proneural), Codel (Codeletion), Noncodel (Non-codeletion), Methyl (Methylated), Unmethyl (Unmethylated), NA (Not available), HCK (Hemopoietic cell kinase, representing immune activities of the monocyte-macrophage lineage), LCK (Lymphocyte-specific kinase, representing T-cell immunity), MHC I (Major histocompatibility complex I, representing presentation of intracellular antigens), MHC II (Major histocompatibility complex II, representing activities of antigen-presenting cells), IgG (representing B-cell immunity), STAT1 (Signal transducer and activator of transcription 1, representing interferon signal transduction), IFN (Interferon, representing interferon-induction and interferon-response). * indicates *p* value < 0.05, **indicates *p* value < 0.01, *** indicates *p* value < 0.001, **** indicates *p* value < 0.0001.

**
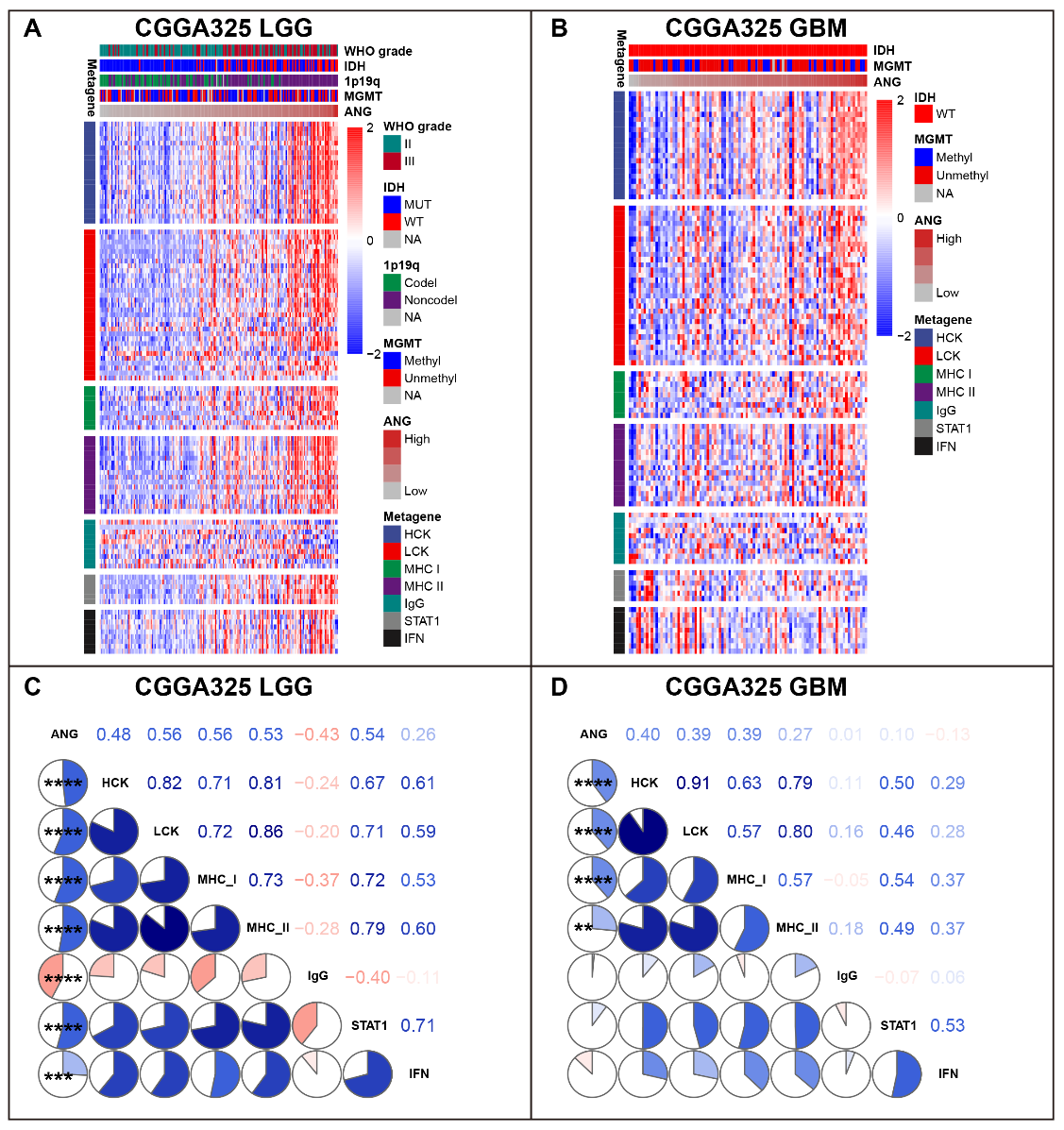
**

**Figure S6.** Gene Sets Variation Analysis (GSVA) of ANG-related inflammatory activities in CGGA325 dataset. (A) Gene heatmap of different inflammatory activities arranged by ANG expression in CGGA325 lower-grade glioma. (B) Gene heatmap of different inflammatory activities arranged by ANG expression in CGGA325 glioblastoma. (C) Intercorrelation between ANG and seven metagenes in CGGA325 lower-grade glioma. (D) Intercorrelation between ANG and seven metagenes in CGGA325 glioblastoma. LGG (Lower grade glioma), GBM (Glioblastoma), MUT (Mutation), WT (Wildtype), CL (Classical), ME (Mesenchymal), PN (Proneural), Codel (Codeletion), Noncodel (Non-codeletion), Methyl (Methylated), Unmethyl (Unmethylated), NA (Not available), HCK (Hemopoietic cell kinase, representing immune activities of the monocyte-macrophage lineage), LCK (Lymphocyte-specific kinase, representing T-cell immunity), MHC I (Major histocompatibility complex I, representing presentation of intracellular antigens), MHC II (Major histocompatibility complex II, representing activities of antigen-presenting cells), IgG (representing B-cell immunity), STAT1 (Signal transducer and activator of transcription 1, representing interferon signal transduction), IFN (Interferon, representing interferon-induction and interferon-response). **indicates *p* value < 0.01, **** indicates *p* value < 0.0001.

**
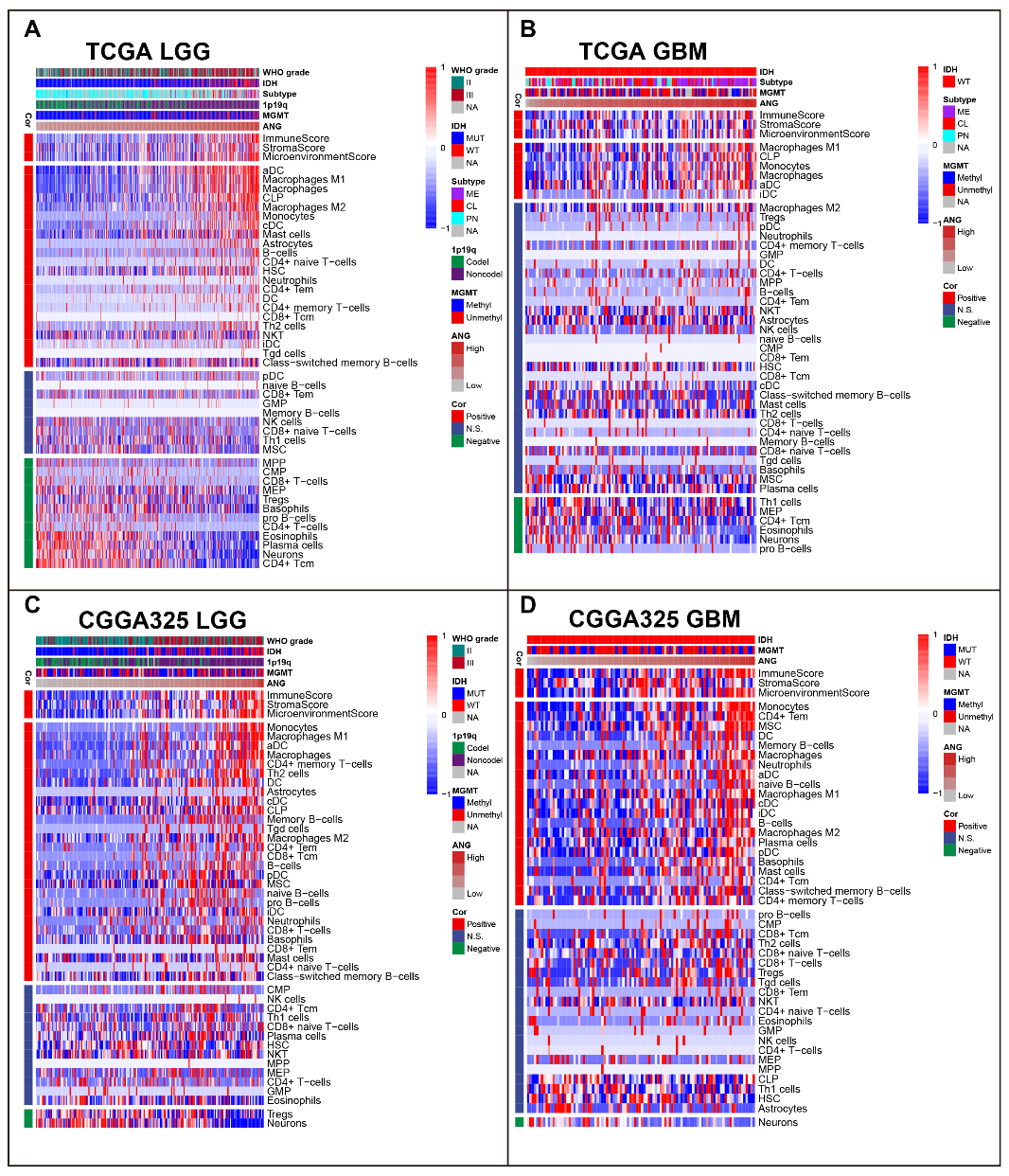
**

**Figure S7.** Relationship between ANG and immune cell subpopulations in TCGA and CGGA325 dataset. (A) Correlation between ANG and immune cell subpopulations in TCGA lower-grade glioma. (B) Correlation between ANG and immune cell subpopulations in TCGA glioblastoma. (C) Correlation between ANG and immune cell subpopulations in CGGA325 lower-grade glioma. (D) Correlation between ANG and immune cell subpopulations in CGGA325 glioblastoma. LGG (Lower grade glioma), GBM (Glioblastoma), MUT (Mutation), WT (Wildtype), CL (Classical), ME (Mesenchymal), PN (Proneural), Codel (Codeletion), Noncodel (Non-codeletion), Methyl (Methylated), Unmethyl (Unmethylated), NA (Not available), Cor (Correlation), N.S. (Not significant), DC (Dendritic cells), aDC (Activated dendritic cells), cDC (Conventional dendritic cells), iDC (Immature dendritic cells), pDC (Plasmacytoid dendritic cells), MSC (Mesenchymal stem cells), NKT (Natural killer-like T cells), CLP (Common Lymphoid Progenitor), Tcm (Central memory T cells), Tem (Effector memory T cells), MPP (Multipotent progenitors), NK (Natural killer), CMP (Common myeloid progenitor), Tgd (T gamma delta cells), Tregs (Regulatory T cells), HSC (Hematopoietic stem cells), MEP (Megakaryocyte-erythroid progenitor), GMP(Granulocyte-macrophage progenitor).
